# Over-Expression of a Wheat Late Maturity Alpha-Amylase Type 1 Impact on Starch Properties During Grain Development and Germination

**DOI:** 10.3389/fpls.2022.811728

**Published:** 2022-03-29

**Authors:** Qin Zhang, Jenifer Pritchard, Jos Mieog, Keren Byrne, Michelle L. Colgrave, Ji-Rui Wang, Jean-Philippe F. Ral

**Affiliations:** ^1^Agriculture and Food, Commonwealth Scientific and Industrial Research Organisation (CSIRO), Canberra, ACT, Australia; ^2^Triticeae Research Institute, Sichuan Agricultural University, Chengdu, China; ^3^Southern Cross Plant Science, Southern Cross University, Lismore, NSW, Australia; ^4^Agriculture and Food, Commonwealth Scientific and Industrial Research Organisation (CSIRO), St Lucia, QLD, Australia

**Keywords:** late maturity alpha-amylase, germination, wheat, dormancy, starch, sprouting

## Abstract

The hydrolysis of starch is a complex process that requires synergistic action of multiple hydrolytic enzymes, including α-amylases. Wheat over-expression of *TaAmy1*, driven by seed specific promoter, resulted in a 20- to 230-fold total α-amylase activity in mature grains. Ectopic expression of *TaAmy1* showed a significant elevated α-amylase activity in stem and leaf without consequences on transitory starch. In mature grain, overexpressed TaAMY1 was mainly located in the endosperm with high expression of *TaAmy1*. This is due to early developing grains having effect on starch granules from 18 days post-anthesis (DPA) and on soluble sugar accumulation from 30 DPA. While accumulation of TaAMY1 led to a high degree of damaged starch in grain, the dramatic alterations of starch visco-properties caused by the elevated levels of α-amylase essentially occurred during processing, thus suggesting a very small impact of related starch damage on grain properties. Abnormal accumulation of soluble sugar (α-gluco-oligosaccharide and sucrose) by TaAMY1 over-expression reduced the grain dormancy and enhanced abscisic acid (ABA) resistance. Germination study in the presence of α-amylase inhibitor suggested a very limited role of TaAMY1 in the early germination process and starch conversion into soluble sugars.

## Introduction

Alpha-amylase (EC 3.2.1.1) has received significant attention from the international wheat community due to its role in germination and the malting process and its involvement in two of the main grain quality defects, namely, pre-harvest sprouting (PHS) and late maturity alpha-amylase (LMA) (Mares and Mrva, 2014). Alpha-amylase belongs to the glycosyl hydrolase (GH) family GH13 (Henrissat, 1991; Majzlova et al., 2013) and catalyses random cleavages of α-1,4-glucosidic bonds in starch, glycogen, oligosaccharides, or polysaccharides to produce reducing sugars (Kandra et al., 2002). In wheat, four isoforms (TaAMY1, TaAMY2, TaAMY3, and TaAMY4) have been reported and well catalogued (Ainsworth et al., 1985; Whan et al., 2014; Ral et al., 2016). The *TaAmy1* locus is a multigene subfamily located on the long arm of chromosome 6, encoding the high pI α-amylase (Gale et al., 1983; Gale and Ainsworth, 1984). In wheat grain, α-amylases are primarily produced during the first stage of germination to degrade endosperm-located starch and to fuel seedling establishment (Ma et al., 2017). When grain starts germinating, dormancy is broken down and the level of abscisic acid (ABA), the key regulating hormone preventing germination, drops (Jacobsen, 1973; Yomo and Varner, 1974). Following the uptake of water (imbibition), the seed goes through a range of metabolic changes which contribute to the eventual emergence of the embryo (Nonogaki et al., 2010). During these early phases of germination, the processes driving germination are heterotrophic, relying heavily on stored resources (Plaxton, 1996). Synthesis of α-amylase occurs in the aleurone layer to initiate starch reserve mobilisation (Chandler et al., 1984; Chandler and Robertson, 1999; Gubler et al., 2002). The α-amylase production is accelerated by gibberellic acid (GA) (Higgins et al., 1976; Gubler et al., 2005). Germination studies have shown that TaAMY1 is primarily generated and released during the first 48–72 h of imbibition, followed by TaAMY2 from 96 h of imbibition (Radchuk et al., 2009). The rationale behind this orderly release remains unknown, but Mieog et al. (2017) suggested that different isoforms of α-amylase may preferentially act on different substrates produced during starch hydrolysis. This hypothesis was corroborated by preliminary structural and functional studies of barley equivalent α-amylases HvAMY1 and HvAMY2, suggesting different affinities of the two enzymes for different substrates (Robert et al., 2003; Cockburn et al., 2015). Recently, Zhang et al. (2021) characterised the impact of overexpressed TaAMY2 on starch degradation during grain development and germination. This research suggested that TaAMY2, and α-amylase in general, is not required to break down starch in the first stage of germination. However, the study was focused on TaAMY2, which is not the primary α-amylase isoform released during the first stage of germination. Thus, the role and impact of TaAMY1 on the early stage of germination remains unknown.

TaAMY1 is well known for its involvement in germination but is also one of the main enzymes associated with major wheat grain quality defects. When grains have very low dormancy or if that dormancy is broken down due to environmental conditions such as cold and rain events, grains can germinate on heads before ripening. This phenomenon, called PHS, leads to the production and release of the entire enzymatic set for germination, including xylanases, proteases, and high levels of two α-amylases (TaAMY1 and TaAMY2) (Mares and Mrva, 2014). Excess of enzymes associated with the loss of grain compositional integrity leads to a substantial reduction of marketability of wheat flour and flour-based products (Gubler et al., 2005; Martinez et al., 2018).

On the other hand, LMA is characterised by stochastic (random) accumulation of a single type of α-amylase (high pI α-amylase, TaAMY1) in the aleurone layer cells (Mrva et al., 2006; Mares and Mrva, 2008). Despite being studied for almost 20 years, the mechanism underlying LMA occurrence remained poorly understood until very recently when Derkx et al. (2021) brought new insights on LMA occurrence. The combination of genetic and environmental conditions causing LMA initiation is still to be elucidated. It has been assumed that elevated levels of α-amylase partially digest starch granules during grain maturation, leading to the reduction of flour baking properties. For over 60 years, starch damage in wheat grains has been assessed by a flour viscosity test, namely, the Falling Number (FN) test, that now accepted as the international standard measure for grain quality (Perten, 1964). While the impact of PHS has been clearly demonstrated (Olaerts et al., 2016), the impact of TaAMY1 accumulation alone on grain composition and starch integrity remains a source for discussion. Studies have demonstrated that elevated levels of α-amylase in wheat grain did not impact baking or noodle quality (Ral et al., 2016, 2017). An additional recent study also showed that LMA had very minor impacts on starch properties (Neoh et al., 2020). It was previously assumed that the physical separation of α-amylase from the starch meant there would be no interaction or effect until milling. However, studies to date have involved populations with a limited range of LMA-related low falling numbers (Newberry et al., 2018) or very small sample set with low replication (Neoh et al., 2020), making comparative study on the impact of TaAMY1 on starch properties cumbersome. Due to the difficulty of generating sufficient biological material concomitantly affected by LMA in the same genetic background, the comparative study on the impact of TaAMY1 on starch properties have not been performed to date. Therefore, the impact of TaAMY1-LMA on grain starch integrity and overall flour quality remains unclear.

The aims of this study were to: (1) examine the impact of increasing TaAMY1 in the endosperm during grain development on starch structure and flour properties starch granules and (2) determine the impact of elevated levels of TaAMY1 on germination and dormancy.

This manuscript describes an approach where the main LMA wheat α-amylases (*TaAmy1*) was specifically overexpressed in wheat grain and its consequences on grain properties and starch functionality was studied.

## Materials and Methods

### Vector Construction and Wheat Transformation

A wheat *TaAmy1* was amplified from cDNA prepared from developing grain 20 days post-anthesis (DPA) of variety Chara. The opening reading frame (ORF) of *TaAmy1* (nt 1–1284) showed strong similarity (Identity: 86.41%) with GenBank accession no. KKY368729.1 (Supplementary Figure 1A). Sequence comparison using the wheat Ensembl Plant search engine indicated that the cDNA-translated protein sequence had 97.2% identity with Chinese Spring TaAMY1 TraesCS6A02G319300 located on chromosome 6A. The ORF was cloned into pCXBx17N (Supplementary Figure 1B) in sense orientation and under control of the grain specific high molecular weight glutenin Bx17 promoter according to the method described by Whan et al. (2014). Transformation was carried out using the method of Richardson et al. (2014) on immature embryos of the wheat variety Fielder.

### Plant Growth and Sampling

All plants were grown in glasshouses at the Commonwealth Scientific and Industrial Research Organisation (CSIRO) Black Mountain Science and Innovation Park, Canberra, Australia Capital Territory, Australia, under natural light on a diurnal temperature cycle of 14/20°C as described by Ral et al. (2012). Plants were automatically watered at a rate equivalent of 10 mm of water every 3 days. For molecular analysis and vegetative characterisation, biological tissue samples were collected from three individual plants for each event grown simultaneously in the same glasshouse.

Developing grains (dissected where appropriate) and other tissue were sampled at appropriate time points (noon for developing grain, sunset, and sunrise for circadian rhythm leaf tissue), snap-frozen in liquid nitrogen, and stored at −80°C until time of analysis.

For grain compositional analysis, grains from several T4 and T5 generation plants for each independent event and negative controls were grown simultaneously in the same glasshouse. Stability of the copy number was assessed by PCR and grains were harvested, bulked, and processed for analysis.

For germination assay, grains were sampled on head at physiological maturity or left on tagged spikes until harvest as appropriate. Grains were sampled from single heads and dried off in a 38°C oven, hand threshed, and stored at −20°C.

### Construct Copy Number

Genome DNA was extracted from the T2 to T4 generation young leaves according to the protocol described by Whan et al. (2014). Construct copy number was estimated using Real-time PCR reactions performed as per Mieog et al.’s (2013) method. Primers for the Nopaline Synthase (NOS) selectable marker transgene and the Epsilon Cyclase gene from Genome A (EC A) are presented in Supplementary Table 1, of which the latter was used as a reference gene. The real-time PCR reactions were run in PikoReal 96 PCR (Thermo, Finland) and included 10 μl SensiMix SYBR green with Fluorescein, 5 μl primer mix, and 5 μl DNA template (5–10 ng/μl).

### Milling and Starch Extraction

Wheat grain from the Bx17A1OE lines and their negative segregants (NC) were milled into wholemeal flour using an UDY Cyclone Sample Mill (UDY Corporation, Fort Collins, CO, United States) fitted with a 0.5 mm screen and without any prior moisture conditioning.

The dried developing grains, germinated grains, and leaf samples were ground using a hammer mill (ESPE GmbH and Co. KG D-82229 Seefeld, Germany).

Starch extraction was performed according to the method of Regina et al. (2004) with some modifications. Starch slurry was first sieved through 200 μm then 100 μm screens. Tailings were removed using 90% percoll (v/v) followed by three final water washes, centrifugation, and freeze drying.

### Total α-Amylase Activity Assay

Total α-amylase activity was measured on 10 mg wholemeal or leaf extracts using the CERALPHA kit (Megazyme International Ireland, Bray Business Park, Bray, Co., Wicklow, Ireland) with the manufacturer’s protocol adapted for flat-bottom 96-well microplate and using SPECTROstar Nano Microplate Reader (BMG LABTECH, Morninington, Victoria, Australia). The results displayed are the mean of three independent assays of three biological replicates.

### Damaged Starch

Damaged starch content was determined on 10 mg wholemeal by Starch Damage Assay kit (Megazyme International Ireland, Bray Business Park, Bray, Co., Wicklow, Ireland), following a 96-well plate format-adapted manufacturer’s protocol and with 20 μl of the supernatant for measurement. Analyses were performed in three technical replicates.

### Total Starch

Total starch was determined using a modified Total Starch Assay Kit (Megazyme) on a triplicate 10 mg of wholemeal or dried leaf. One millilitre of 80% ethanol (v/v) was added and each sample was incubated using a BioShake at 80°C and 1,000 rpm for 20 min. After centrifugation at 13,000 *g* for 10 min, the supernatant was carefully discarded in order to remove soluble sugars. Centrifugation at 13,000 *g* for 10 min and the removal of supernatant was repeated before the pellet was resuspended in 40 μl of 50% (v/v) ethanol and 400 μl of 50 mM Mops buffer (pH 7.0). Ten microlitres of thermostable α-amylase were added and incubated at 100°C and 1,000 rpm for 10 min. Then, 600 μl of sodium acetate buffer (pH 4.5) and 10 μL of amyloglucosidase were added and vortexed for 5 s. This was followed with a 50°C incubation using a BioShake at 1,000 rpm for a minimum of 60 min. After centrifugation at 13,000 *g* for 10 min, 20 μl aliquots in duplicate with appropriate dilutions were transferred to 96-well flatted bottom plate with 180 μl of GOPOD. Absorbance was read using a SPECTROstar Nano Microplate Reader (BMG LABTECH, Australia) at 510 nm after a 20 min incubation at 50°C. The 1 mg/ml of glucose standard from the kit was used to generate a standard curve.

### Content of α-Gluco-Oligosaccharide, Free Glucose, and Free Fructose

Triplicate 10 mg samples were extracted using 400 μl of 80% (v/v) ethanol for 10 min in a BioShake at 100°C and 1,000 rpm. The extraction was performed three times and all ethanol soluble fractions were pooled after centrifugation. Total soluble sugar was determined using anthrone reagent (0.2% anthrone, in 70% H_2_SO_4_ v/v), and 1 mg/mL of glucose was used for the standard curve (Whan et al., 2014). Free glucose and fructose were measured according to the method described by Campbell et al. (1999) and sucrose was determined as described by Birnberg and Brenner (1984). The soluble α-gluco-oligosaccharide was measured by taking 200 μl of the ethanol soluble fraction dried in a DNA SpeedVac Concentrator (DNA120-230, Thermo Fisher, Waltham, Massachusetts, United States) and then using the Total Starch Assay Kit (Megazyme) as described above.

### Protein Extraction and Digestion

Triplicate biological samples from wholemeal flour from α-amylase TaAMY1 overexpression positive lines (Bx17A1OE-4; D1, D2, and D3) were prepared and digested as described by Whan et al. (2014). The TaAMY2 of UA2OE lines (A1, A2, and A3) and their isogenic negative controls (A4N, A5N, and A6N) were included as controls (Zhang et al., 2021) (Supplementary Table 2).

### LC-MS/MS Analysis and Development of Multiple Reaction Monitoring Assay for α-Amylase (TaAMY1)

The digested peptides (4 μl) from the positive overexpression lines (Bx17A1OE-4; D1, D2, and D3) were chromatographically separated on an Ekspert nanoLC415 (Eksigent, Dublin, CA, United States) and directly coupled to a 6,600 TripleTOF MS (SCIEX, Redwood City, CA, United States) following the method described in MacLean et al. (2010); Colgrave et al. (2014)Colgrave et al. (2017), and Whan et al. (2014). The peptides were desalted for 5 min on a ChromXP C18 (3 μm, 120 Å, 10 mm × 0.3 mm) trap column at a flow rate of 10 μl/min 0.1% FA, and separated on a ChromXP C18 (3 μm, 120 Å, 150 mm × 0.3 mm) column at a flow rate of 5 μl/min at 30°C. A linear gradient from a 3–25% solvent B was employed for 68 min followed by: 5 min from 25% B to 35% B; 2 min 35% B to 80% B; 3 min at 80% B, 80-3% B, 1 min; and 8 min re-equilibration. The solvents were as follows: (A) 5% DMSO, 0.1% FA, 94.9% water; and (B) 5% DMSO, 0.1% FA, 90% acetonitrile, 4.9% water. The instrument parameters were as follows: ion spray voltage 5,500 V, curtain gas 20 psi, GS1 15 psi, GS2 15 psi, and heated interface at 150°C. Data were acquired in information-dependent acquisition (IDA) mode comprising a time-of-flight -MS survey scan followed by 30 MS/MS, each with a 50 ms accumulation time. First stage MS analysis was performed in positive ion mode, mass range m/z 400-1,250, and 0.25 s accumulation time. Tandem mass spectra were acquired on precursor ions >150 counts/s with charge state 2-5 and dynamic exclusion for 15 s with a 100 ppm mass tolerance. Spectra were acquired over the mass range of 100-1,500 m/z was counted every 24 h over the time of the experiment. using the manufacturer’s rolling collision energy capillary electrophoresis (CE) based on the size and charge of the precursor ion.

ProteinPilot™ 4.1.46 software (SCIEX) with the Paragon Algorithm was used for the identification of proteins. Tandem mass spectrometry data were searched against *in silico* tryptic digests of Poaceae proteins of the Uniprot database (version 2017/05; 2,874,344 sequences) appended with the common repository of adventitious proteins (cRAP database). All search parameters were defined as iodoacetamide modified with cysteine alkylation, with trypsin as the digestion enzyme. Modifications were set to the “generic workup” and “biological” modification sets were provided with this software package, which consisted of 126 possible modifications, for example, acetylation, methylation, and phosphorylation. The generic workup modifications set contains 51 potential modifications that may occur as a result of sample handling, for example, oxidation, dehydration, and deamidation. Peptides with missed cleavages were identified but were not included in subsequent MRM analysis.

Multiple reaction monitoring (MRM) transitions were determined for each peptide where the precursor ion (Q1) m/z was based on the size and expected charge, and the fragment ion (Q3) m/z values were predicted using known fragmentation patterns and/or the data collected in the identification workflows. In the preliminary analyses, five peptides were selected and, additionally, modified forms of two peptides were monitored as these contained methionine which could be subject to variable levels of oxidation. The final MRM method consisted of eleven non-modified peptides for TaAMY1 (Supplementary Figure 2) with three best responding MRM transitions per peptide, where in the peak area of the three MRM transitions were summed (Supplementary Table 3).

Reduced and alkylated tryptic peptides were analysed on a 6,500 QTRAP mass spectrometer (SCIEX) equipped with a TurboV ionisation source operated in positive ion mode. Samples (5 μl) were chromatographically separated on a Shimadzu Nexera Ultra High Pressure Liquid Chromatography (UHPLC; Shimadzu) using a Phenomenex Kinetex C18 (2.1 mm × 10 cm) column with a linear gradient of 5–45% acetonitrile for 10 min with a flow rate of 400 μl/min. The eluent from the high pressure liquid chromatography (HPLC) was directly coupled to the mass spectrometer. Data were acquired and processed using Analyst™ 1.6.3 software. Quantitation of the α-amylase tryptic peptides was achieved using MRM experiments and a 40 s detection window for each MRM transition and a 0.3 s cycle time. Peptides were fragmented in the collision cell with nitrogen gas using collision energy dependent on the size and charge of the precursor ion. The MS parameters were ionspray voltage (IS) 5,500 V, curtain gas 35, GS1 40, GS2 50, source temperature of 500°C, declustering potential (DP) set to 70, and entrance potential (EP) set to 10. Peaks were integrated using Skyline (MacLean et al., 2010) wherein all three MRM transitions were required to co-elute at the same retention time (RT) with a signal-to-noise (S/N) > 3 for detection and a S/N > 5 for quantitation and graphed using Excel (version 1908).

### Fructan Content

The fructan was extracted using distilled water as described by Verspreet et al. (2012) with some modifications. Triplicate 5 mg samples were suspended in 1 ml distilled water and placed at 80°C for 30 min in a Bioshake at 1,000 rpm. After cooling to room temperature, the wheat/water mixture was centrifuged at 13,000 rpm for 10 min. The total fructan was measured according to Fructan HK Procedure (Megazyme, AOAC method 999.03 and AACC Method 32.32.01).

### Isolation of Tissue in Mature Grain

Sixty grains per line were used for tissue isolation according to the method described by Barrero et al. (2013) and Daneri-Castro and Roberts (2016). The husk and embryo from mature grains were isolated and collected after imbibition with 40 ml water at 4°C for 4 h. The crease tissue was removed from deembryonated grains and the remaining tissues were imbibed in 10 ml of Milli-Q water containing 150 μg/ml Cefotaxime and 50 μg/ml nystatin for 16 h in *Petri* dishes at 4°C. After imbibition, aleurone layer and starchy endosperm were isolated with a scalpel. All tissues were snap frozen in liquid nitrogen and freeze-dried in VirTis BenchTop Pro freeze dryer (SP Scientific).

### Late Maturity Alpha-Amylase ELISA Test

High-pI α-amylase was determined by ELISA test as described by Barrero et al. (2013) and Mieog et al. (2017). Three replicates of 5 mg of wholemeal or isolated tissue and 10 mg of leaf or stem were used for the assay.

### Falling Number

The FNs of all lines were performed on a FN instrument FN1000 (Perten Instruments, Hägersten, Sweden) using 7 g of wholemeal per sample and in triplicate with each sample mixed with 25 ml Milli-Q water according to the manufacturer’s instructions.

### Rapid Viscosity Analysis

Starch pasting properties were determined using RVA 4500 (Perten Instruments Australia Pty Ltd., Sydney, NSW, Australia) as described by Ral et al. (2016). The addition of 0.5 ml of 10% silver nitrate (v/v) was used for inhibiting α-amylase activity.

### Differential Scanning Calorimetry

Differential scanning calorimetry analysis of isolated starch was performed with a Perkin DSC 8000 (PerkinElmer, Pty. Ltd., Melbourne, Victoria, Australia). Approximately 60 mg of starch was premixed with water at a ratio of 1:2 and weighed into aluminium DSC pans, sealed, and left to equilibrate overnight, following the protocol described in Ral et al. (2016). Results displayed are the mean of three independent assays.

### Particle Size Distribution

Malvern Mastersizer 3000 with Hydro MV wet sample dispersion unit (Malvern Instruments, Ltd., Malvern, United Kingdom) was used to determine the starch granule size distribution by laser diffraction according to the manufacturer’s instructions. Average granule sizes were recorded as circular equivalent diameters (CE diameter) at 10% (D10), 50% (D50), and 90% (D90) of the starch wet sample volume. The B granule content was taken as the proportion of granules with a diameter of less than 10.1 μm.

### Morphometric Analysis of Starch Granule Size and Shape Distribution

Evenly dispersed dry samples of isolated starch were analysed for morphometric differences in size and shape distributions via automated static image analysis with the Morphologi 4 ID (Malvern Panalytical-ATA Scientific, Sydney, NSW, Australia). An average of 2,500 individual granules were imaged per sample in triplicate. In order to make comparisons with the laser diffraction results from the Mastersizer, the same size categories of CE diameter were used and expressed as a percentage of the total number of starch granules imaged. This data was also converted to spherical equivalent (SE) volume and expressed as a percentage of the total volume of starch granules imaged.

### Amylose Content

Amylose content was measured following small-scale iodine adsorption method on flour as described in Regina et al. (2004). Standard samples containing 0–100% amylose were used to generate a standard curve. The absorbance of the test samples were converted to percentage amylose using a regression equation derived from the standard samples.

### Scanning Electron Microscope

Four grains of each collection were fixed and washed in 70% ethanol (v/v) for 2 h. Then, all grains were sectioned transversely into 1 mm slices with a razor blade and then freeze-dried. The microstructure of isolated starch was observed using scanning electron microscope (SEM) as described by Whan et al. (2014).

### Germination Assay

Germination assays were performed as described by Jacobsen et al. (2013). Grains (T5 generation) from T4 plants were harvested at physiological maturity and 4 weeks after physiological maturity on the mother plant. The protocol for harvest followed Gubler et al. (2008) except for details outlined as follows. Harvested grains were dried at 37°C for 24 h with low humidity and were then stored at −20°C. Grains were imbibed on 9 cm plastic *Petri* dishes containing 0.3% agarose (w/v) and MilliQ water. Plates were incubated at 20°C in the dark. Coleorhiza emergence (CE) was counted every 24 h over the time of the experiment.

Abscisic acid [ABA; species “(±) –”, Sigma-Aldrich, CAS. No. 14375-45-2] was prepared by dissolving 7.93 mg of dry powder in 260 μl of absolute ethanol, followed by dilution with sterilised MilliQ water to a 2 mM stock solution concentration. ABA resistance was tested by addition of ABA (0, 20, 50, 100, and 150 μM) from a 2 mM stock solution to a sterilised 0.3% agarose (w/v and adjusted at pH 6) according to Kondhare et al. (2012). Acarbose (Sigma-Aldrich, CAS. No. 56180-94-0) (Oudjeriouat et al., 2003) was added to sterilised 0.3% agarose to give the final desired concentration of acarbose (0 μM, 200 μM, 500 μM, 1 mM, and 1.5 mM) from a 2 mmol/ml stock solution. The acarbose concentration of 1.5 mM was the minimum concentration to completely inhibit α-amylase in wheat grains. Germination experiment included either three replicates of 20 whole grains of Bx17A1OE and negative control per 9 cm *Petri* dishes and placed crease down or 20 halved grains placed cut side down in 9 cm *Petri* dishes with 0.3% agarose containing acarbose and/or ABA.

### Statistical Analysis

All data were expressed as mean ± standard error (SE) of at least three replicates. One-way analysis of variance (ANOVA) was performed on all data to indicate significant difference (*p*-values < 0.05). All figures were prepared using GraphPad Prism 8.0.

## Results

### High TaAMY1 Content in Transgenic Wheat

To investigate the impact of a high content of TaAMY1 on grain quality, a vector carrying *TaAmy1* cDNA under control of Bx17 glutenin promoter and NOS terminator (selected marker) was transformed into wheat (Supplementary Figure 1) (Ral et al., 2012). Eight hygromycin resistant *TaAmy1* (Bx17A1OE) wheat T0 plants were selected by PCR using construct-specific primers (Supplementary Table 1) following *Agrobacterium tumefaciens*-mediated transformation of immature embryos.

From T1 generation, the number of insertions was traced (Supplementary Table 4). Due to large variations in copy number and total α-amylase activity, five transgenic lines were isolated from different T0 plants, labelled Bx17A1OE1.2 (8 copies), Bx17A1OE2.2 (4 copies), Bx17A1OE3.1 (4 copies), Bx17A1OE4.1 (10–18 copies), and Bx17A1OE4.6 (4 copies). The first three lines were generated from different transformation events. Particularly, Bx17A1OE4.1 and 4.6 were from different regenerated shoots from the one Agrobacterium treated embryo. Two negative segregants were selected and labelled 2-8NS and 4-6NS. An LMA-affected variety (Chara) containing aleurone specific TaAMY1 accumulation was added for the preliminary characterisation as a reference. As this variety was not grown under the same conditions, it was decided to be included for the α-amylase localisation experiment only. Copy number was monitored from T2 to T4 generation (Supplementary Table 4) and strong correlations were detected between copy number and total α-amylase activity in T2 (*R*^2^ = 0.8435) and T4 grains (*R*^2^ = 0.7212) (Supplementary Figure 3).

**FIGURE 1:**
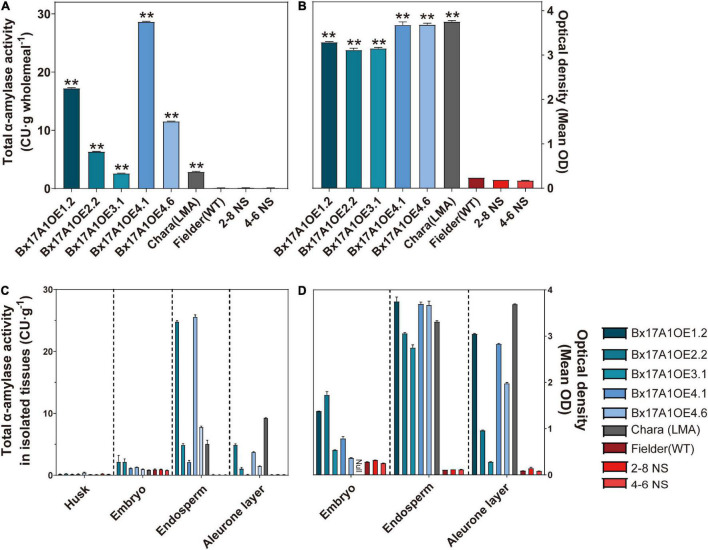
Total α-amylase activity and late maturity alpha-amylase (LMA)-ELISA test in wholemeal flour and TaAMY1 tissue location. **(A)** Total α-amylase activity is expressed in Ceralpha Units (CU) of per gramme wholemeal. **(B)** TaAMY1 protein presence in wholemeal flour is expressed by optical density (OD) per 5 mg wholemeal. Total α-amylase activity **(C)** and TaAMY1 protein **(D)** in isolated embryo, endosperm, and aleurone layer. Three technical replicates were performed with spherical equivalent (SE) displayed. Bx17A1OE lines were displayed as blue, Chara (LMA) was displayed as grey, and negative controls were displayed as red. *p*-values are indicated as follows **p < 0.01.

These five independent events showed a strong increase in total α-amylase activity (from 20- to 230-fold) compared to Fielder (wild type, WT) and the two negative segregants (Figure 1A) with the 20-fold increase (in the BX17AOE 3.1 line) that had similar amplitude to those seen in the LMA-affected line.

An LMA-TaAMY1-specific ELISA test showed a high accumulation of TaAMY1 protein in the Bx17A1OE lines. The results showed that the LMA-affected Chara response ranged from 3.17 to 3.68 OD, while Fielder and two negative segregants had relatively low levels of TaAMY1. Particularly, with an average of 0.19 (Figure 1B). Finally, analysis of total proteins extracted from Bx17A1OE dry grains using mass spectrometry detected nine TaAMY1 peptides covering the entire protein sequence in the Bx17A1OE lines. No TaAMY1-specific peptides were detected in NC (Supplementary Figure 4). No TaAMY2 and TaAMY3 specific peptides were detected in either positive lines or negative controls. These results strongly suggested that the elevated level of total α-amylase activity detected in the Bx17A1OE lines was uniquely due to the over-expression of TaAMY1 (Supplementary Figure 2 and Supplementary Table 3).

### High TaAMY1 Content With No Impact on Transitory Starch

**FIGURE 2:**
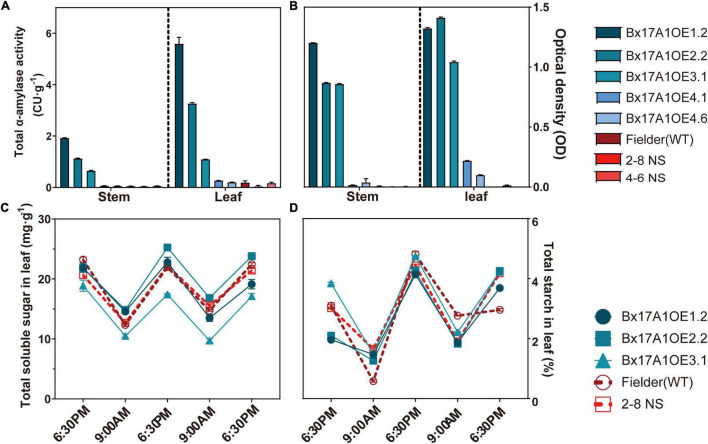
Effect of TaAMY1 overexpression on stem and leaf sugar composition. Total α-amylase activity **(A)** and TaAMY1 protein content expressed by optical density **(B)** in stem and leaf. Total soluble sugar **(C)** and total starch **(D)** from dry leaf at sunrise and sunset for three consecutive days. Three technical replicates were performed with SE displayed.

The Bx17 promoter is grain-specific, but has shown, in some cases, leakage in other tissues (Whan et al., 2014). Total α-amylase activity and ELISA test were assessed to ensure that TaAMY1 activity did not impair starch accumulation in vegetative tissues. Fielder and two negative segregants showed a low level of α-amylase activity in both stem and leaf with 0.04 ± 0.01, 0.04 ± 0.00, and. 005 ± 0.01 CU⋅g^–1^ in stem, and 0.19 ± 0.07, 0.04 ± 0.04, and 0.16 ± 0.04 CU⋅g^–1^ in leaf, respectively (Figure 2A). Compared to Fielder and negative segregants, only Bx17A1OE1.2, Bx17A1OE2.2, and Bx17A1OE3.1 showed a significant elevated α-amylase activity in stem and leaf. The ELISA test verified that only these three positive lines showed an increase in TaAMY1 accumulation in stem and leaf compared to Fielder and the negative segregants (Figure 2B). These three high α-amylase activity lines had a similar soluble sugar and total starch profile compared to Fielder and one negative segregant (Figures 2C,D). This suggested the unexpected presence of TaAMY1 in vegetative tissue had no impact on leaf starch accumulation.

### *TaAmy1* Transcript and Engineered TaAMY1 Profiling During Grain Development

To determine the expression pattern of *TaAmy1* during grain development, real-time PCR (qRT-PCR) was used for *TaAmy1* transcript from tissue extracted at 3 key developmental stages of cellularisation (5 days post anthesis, DPA), starch accumulation (15 DPA), and grain maturation (25 DPA) (Supplementary Figure 5). At the 3 time points, the level of *TaAmy1* transcript in Bx17A1OE1.2, Bx17A1OE3.1, and Bx17A1OE4.1 was consistently higher than the controls, with up to 9.14 times increase compared to negative segregant at 15 DPA, and 100 times higher at 25 DPA.

**FIGURE 3:**
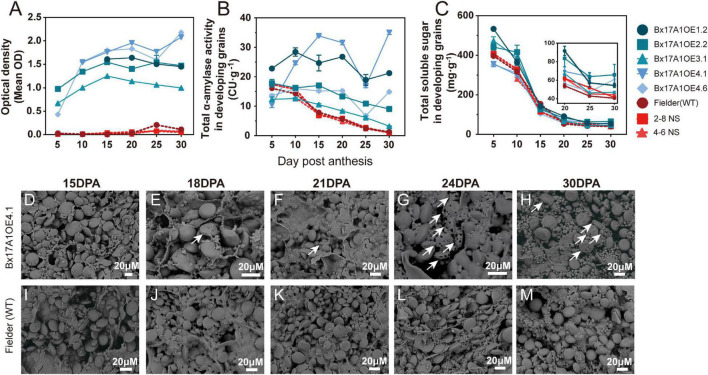
Effect of TaAMY1 overexpression on grain composition and starch morphology during development. TaAMY1 protein content expressed by optical density **(A)** and total α-amylase activity expressed in Ceralpha Units (CU) of per gramme of tissue **(B)**, and total soluble sugar expressed in mg.g tissue^– 1^ **(C)** during grain development. Scanning electron micrographs (SEM) of representative grain cross sections from Bx17A1OE4.1 **(D–H)** and Fielder (wild type, WT) **(I–M)** during key grain development stage. White arrows indicated starch degraded by TaAMY1.

Total α-amylase activity and TaAMY1 protein content were also assessed during grain development (Figure 3). In Fielder and the negative segregants, TaAMY1 protein levels were consistently at the lowest limit of detection during grain development, with a noticeable spike at 25 DPA (Figure 3A). In the Bx17A1OE lines, TaAMY1 protein levels were elevated and increasing from 5 to 15–20 DPA. Then, overall levels plateaued or slightly increased until 30 DPA. Total α-amylase activity in all negative controls decreased from 5 to 30 DPA to reach a very low level (Figure 3B). It is noteworthy that the detected activity in the negative controls might not be due to TaAMY1 as the protein responsible for this α-amylase activity was not detected using the TaAMY1 specific ELISA test.

From anthesis, three Bx17A1OE lines exhibited similar total α-amylase activities to those seen in the negative controls. Although after 10 DPA, the level of total α-amylase activity in these lines decreased less rapidly compared to controls. At 5 DPA, only Bx17A1OE1.2 and Bx17A1OE2.2 showed a significant increase in total α-amylase activity with over 23–35 CU⋅g^–1^ compared to WT (16 CU⋅g^–1^), remaining consistently higher throughout the grain development. While presenting different levels of total activity, the TaAMY1 overexpressing lines consistently showed a relatively high activity at 30 DPA, thus accentuating the difference in total α-amylase activity compared to the negative controls. This elevated level of α-amylase activity was correlated with higher levels of soluble carbohydrate in the Bx17AMYOE lines throughout grain development, but was also more pronounced toward the end of grain development concomitantly with the starch damage and pin hole occurrance (Figure 3C).

### Localisation of TaAMY1 Protein in Transgenic Lines and Late Maturity Alpha-Amylase Wheat

As LMA is defined as an accumulation of TaAMY1 in aleurone during the grain development that has potential impact on starch structure and composition, the localisation of TaAMY1 protein and α-amylase activity between transgenic lines, controls, and an LMA Chara was examined. The seed coat, embryo, endosperm, and aleurone layer were partially isolated from imbibed mature grains. TaAMY1 protein content was primarily in the LMA Chara aleurone layer as expected, but was also detected in the endosperm that translated into higher α-amylase activity in both tissues (Figures 1C,D). As the mechanical separation of aleurone layer from endosperm is cumbersome and requires pre-imbibition of the grain, some partial cross-contamination between the two tissues could be expected. Almost no TaAMY1 ELISA signal or total activity was detected in negative controls. In the Bx17AMY1OE lines, most of the response and associated activity was found in the endosperm and the aleurone layer with α-amylase activity ranging from 2.12 ± 0.15 to 25.5 ± 0.21 CU⋅g^–1^. LMA Chara, in particular, showed 5.03 ± 0.36 CU⋅g^–1^. A higher ELISA signal was detected in the embryos compared to their negative controls, but did not translate to significant increase of activity. The results indicated that the engineered TaAMY1 is present in the endosperm and aleurone, potentially allowing enzymatic attack on starch granules.

Microscopic observation showed signs of degradation as illustrated by pin holes and channels only from 18 DPA onwards in Bx17A1OE lines, while Fielder did not show any visual signs of damage (Figures 3D–M). This observation confirmed the impact of overexpressed TaAMY1 on starch structure during grain development, but occurring at a later developmental stage, suggesting an inability for the enzyme to access starch granules earlier.

### Impact of Starch and Carbohydrate Composition

**FIGURE 4:**
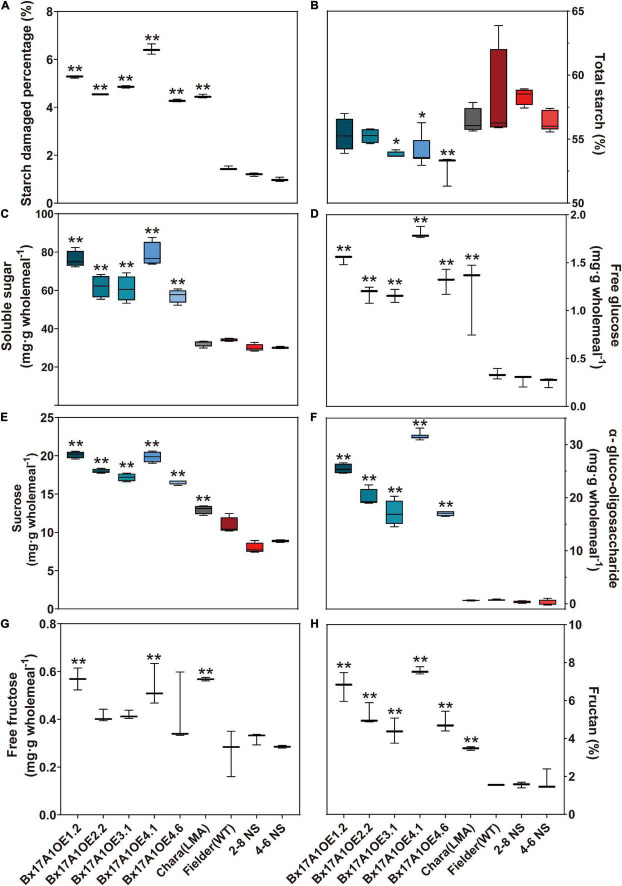
Effect of TaAMY1 overexpression carbohydrate composition in mature grains. Damaged starch **(A)**, starch **(B)**, and fructan content **(H)** are expressed in percentage of wholemeal flour. Total soluble sugar **(C)**, free glucose **(D)**, sucrose **(E)**, α-gluco-oligosaccharide **(F)**, and free fructose **(G)** are expressed in milligramme per gramme wholemeal. *p*-values are indicated as follows *p < 0.05 and **p < 0.01. Error bars represent Standard Error of three replicates.

Damaged starch and carbohydrate composition were measured to investigate the potential impact of excess TaAMY1 on soluble carbohydrate composition in Bx17A1OE lines. The Bx17A1OE lines showed a significant increase in the percentage of damaged starch (4.27–6.42%) compared to negative controls (0.99–1.46%) (Figure 4A). This increase in damaged starch was concomitant to a total starch decrease (∼1–3%) in the Bx17A1OE lines (Figure 4B) and significantly elevated total soluble sugars ranging from 57.09 to 78.66 mg⋅g DW^–1^ in mature grains compared to Fielder and negative segregants (32 mg⋅g DW^–1^ on average) (Figure 4C). Several types of sugar (glucose, sucrose, α-gluco-oligosaccharides, fructan, and fructose) were measured in the five positive lines (Figures 4D–G). Significant increases in α-gluco-oligosaccharides, free glucose, and sucrose were detected in the five positive lines with 43. 2-, 4. 74-, and 1.9-fold increases on average, respectively, compared to the negative controls. Free fructose in the Bx17A1OE lines showed no significant increase compared to Fielder and the negative segregants (Figure 4G). The level of fructan in the positive lines showed a significant increase ranging from 44.1 to 75.7 mg⋅g DW^–1^ compared to Fielder and two negative segregants (15.6 mg⋅g DW^–1^ on average) (Figure 4H).

The granule size distribution and amylose content of isolated starch were examined. No significant differences were observed in starch granule size distribution based on the volume distributions determined by laser diffraction (Supplementary Figure 6A). However, the morphometric image analysis of the dry dispersed starch granules revealed differences in the granule size distributions between the negative controls and transgenic lines. The percentage distributions, based on the number of starch granules imaged clearly, showed three populations of starch granules in Fielder and the negative segregants with size ranges from ≤2, 2 to 10 μm, and >10 μm (Supplementary Figures 6B,C). These size ranges are consistent with and may be categorised as populations of C, B, and A granules, respectively. The number of imaged starch granules below 10 μm or the corresponding calculated spherical equivalent volume of the starch granules in each size class were reduced in the transgenic lines compared to the parental line Fielder and the negative segregants 2-8NS and 4-6NS (which were themselves indistinguishable from each other). The percentage of starch granules below 10 μm in this case were substantially reduced, ranging between approximately 15 and 24%. The reductions in the corresponding calculated volume distributions were much smaller, but still significant between 0.72 and 1.1%. No significant difference was observed for the amylose content (Supplementary Figure 7A).

### Impact on Starch Properties

Viscosity and gelatinisation tests were performed on wholemeal samples to investigate the effect of elevating α-amylase activity on starch gel viscosity. Elevated levels of TaAMY1 in the case of LMA lead to strong reduction of starch viscosity and, consequently, to unacceptable low FN. In the absence of silver nitrate (α-amylase inhibitor), Fielder and the two negative segragants displayed a Rapid Visco Analyser (RVA) profile with high values of peak, trough, and final viscosity ranging between 810.5–1340.3, 81–251, and 196–615 cP, respectively (Table 1 and Supplementary Figure 7B). The Bx17A1OE lines showed significant declines in peak, trough and final viscosity with ranges of 80–277, 12.7–27.7, and 28–39 cP, respectively. LMA-affected Chara still showed a decrease in peak, trough, and final viscosity at 97.7 ± 1.9, 19.7 ± 0.7, and 30.3 ± 0.9, respectively.

**TABLE 1:** Rapid Visco Analyser (RVA) profile and gelatinization properties (DSC).

Samples	Rapid Visco Analyser					Gelatinization endotherm				

	Peak (cP)	Breakdown (cP)	Trough (cP)	Setback (cP)	Final Visc (cP)	Onset temp.	Peak temp.	End temp.	Delta *H*	Falling number (S)
Bx17A1OE1.2	156.3 ± 6.9**	142.3 ± 6.9**	14 ± 0**	14.3 ± 0.7**	28.3 ± 0.7**	56.4 ± 2.1	61.5 ± 0.3	67.5 ± 0.4	5.9 ± 0.2	62 ± 0
Bx17A1OE1.2 + AgNO_3_	3705.7 ± 44.7	2407 ± 36.1	1298.7 ± 12	1619 ± 12.3	2917.7 ± 23.4					
Bx17A1OE2.2	277.3 ± 12.4**	260.7 ± 13**	16.7 ± 0.7**	14 ± 0.6**	30.7 ± 0.9**	57.8 ± 0	60.8 ± 0.1	68 ± 0.9	6.5 ± 0.1	62 ± 0
Bx17A1OE2.2 + AgNO_3_	3901.7 ± 10.4	2576.3 ± 8.8	1325.3 ± 2.7	1633.7 ± 28	2959 ± 25.9					
Bx17A1OE3.1	269 ± 7.5**	241.3 ± 8.1**	27.7 ± 1.2**	11.3 ± 0.9**	39 ± 1.2**	59.7 ± 0.2	63.6 ± 0.8	68.7 ± 2.5	6.5 ± 0.6	66.6 ± 4.6
Bx17A1OE3.1 + AgNO_3_	3504.7 ± 26.5	2344.7 ± 25.8	1160 ± 5	1334.3 ± 13.5	2494.3 ± 8.7					
Bx17A1OE4.1	80.3 ± 3.8**	67.7 ± 4.1**	12.7 ± 0.3**	18.3 ± 0.3**	31 ± 0.6**	59.1 ± 0.3	62.3 ± 0.3	66.5 ± 0.3	5.8 ± 0.3	62 ± 0
Bx17A1OE4.1 + AgNO_3_	3649.3 ± 34	2386.7 ± 15.8	1262.7 ± 18.5	1649 ± 10.5	2911.7 ± 21.7					
Bx17A1OE4.6	268 ± 7**	252 ± 8**	16 ± 1**	15 ± 0**	31 ± 1**	58.6 ± 0.3	61.9 ± 0.3	65.8 ± 0.4	6.1 ± 0.2	62 ± 0
Bx17A1OE4.6 + AgNO_3_	3534 ± 12	2308.5 ± 3.5	1225.5 ± 8.5	1578 ± 20	2803.5 ± 28.5					
Chara (LMA)	97.7 ± 1.9**	78 ± 2.5**	19.7 ± 0.7**	10.7 ± 0.3**	30.3 ± 0.9**	58.5 ± 0.2	62.2 ± 0.2	67.4 ± 0.2	6.2 ± 0.1	62 ± 0
Chara (LMA) + AgNO_3_	3191.7 ± 12.9	1709.3 ± 6.6	1482.3 ± 8.7	1905.7 ± 10.5	3388 ± 13					
Fielder (WT)	810.5 ± 4.5	729.5 ± 6.5	81 ± 2	115 ± 2	196 ± 4	57.6 ± 0.1	61.3 ± 0.5	67.9 ± 0.2	5.9 ± 0.7	228 ± 1.4
Fielder (WT) + AgNO_3_	3994 ± 29	2565.5 ± 8.5	1428.5 ± 20.5	1682 ± 14	3110.5 ± 6.5					
2–8 NS	1340.3 ± 27.5	1112 ± 12.5	228.3 ± 15	367 ± 20.7	595.3 ± 35.7	57.6 ± 0.6	61 ± 0.7	67.3 ± 1.2	6.4 ± 0.4	297 ± 2.8
2–8 NS + AgNO_3_	4362.7 ± 35.8	2812.3 ± 64.4	1550.3 ± 28.7	1796.3 ± 3.5	3346.7 ± 31.9					
4–6 NS	1147.5 ± 35.5	896.5 ± 15.5	251 ± 20	364 ± 31	615 ± 51	57.8 ± 0.5	61.1 ± 0.5	68.1 ± 0.7	6.3 ± 0.2	301.5 ± 6.3
4–6 NS + AgNO_3_	4075.5 ± 8.5	2612.5 ± 1.5	1463 ± 10	1716 ± 19	3179 ± 29					

Rapid Visco Analyser profiles of Bx17A1OE, late maturity alpha-amylase (LMA) wheat and negative controls in presence or absence of the Silver Nitrate (α-amylase inhibitor). Viscosity is expressed in Centipoise (cP). Temperature is expressed in °C in gelatinization endotherm. The enthalpy of gelatinization (ΔH) is expressed in joules per g (J/g). Results displayed are the mean of three independent assays. Asterisks indicate significant differences between positive lines or LMA wheat and every negative control (**p < 0.01).

Addition of silver nitrate drastically increased peak, trough, and final value of the entire set, including Bx17A1OE lines and LMA Chara with peak viscosity, trough, and final viscosities of 3,800, 1,500, and 3,000 cP on average, respectively (Table 1 and Supplementary Figure 7C). The addition of silver nitrate in the Bx17A1OE flours restored more than 90% of the viscosity profile of the Negative controls, suggesting a very limited impact of engineered TaAMY1 on starch viscosity in the developing endosperm prior to addition of water to flours. FN values from Fielder and the two negative segregants were 228, 297, and 301.5 s, respectively (Table 1). FNs of the Bx17A1OE lines and LMA impacted Chara and dropped to the minimal quantitative value measurable on the machine (62 s), confirming the classification of the Bx17A1OE lines as severely sprouted and suitable only as feed grade.

Differential scanning calorimetry (DSC) of purified starch showed no significant difference between Bx17A2OE lines and negative controls (Table 1).

### Reduced Dormancy in Bx17A1OE Lines

**FIGURE 5:**
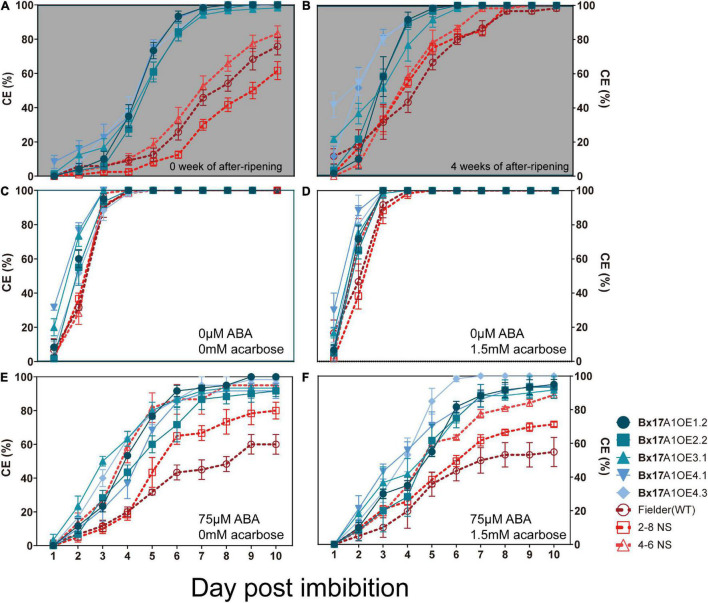
Effect of after-ripening, abscisic acid (ABA) and acarbose on grains germination. Coleorhiza emergence (CE) over days after imbibition for whole grains at physiological maturity **(A)** and 4 weeks after ripening **(B)** in whole grains. CE for half grains at physiological maturity in absence of ABA and acarbose **(C)**, in presence of 1.5 mM of acarbose **(D)**, 75 μM of ABA **(E)** and in presence of both 1.5 mM acarbose and 75 μM ABA **(F)**. Germination rate comparisons between five Bx17A1OE lines (blue) and three negative control (red). The blue circle, square, triangle, inverted triangle, and rhombus were indicated Bx17A1OE1.2, Bx17A1OE2.2, Bx17A1OE3.1, Bx17A1OE4.1, and Bx17A1OE4.6, respectively. Red circle, square and triangle were indicated Fielder, 2-8 NS, and 4-6 NS, respectively. Error bars represent Standard Error of three replicate plates.

Previous studies showed opposite results in terms of impact of TaAMY overexpression on grain germination. Whan et al. (2014) showed a slower germination rate when TaAMY3 was overexpressed. Recently, Zhang et al. (2021) showed an absence of dormancy coupled with ABA resistance when TaAMY2 was overexpressed. It was interesting to analyse the impact of the third isoform of α-amylase on germination. Germination assays using whole or half grains were performed to investigate the effect on dormancy retention. The harvest-ripe grains from Fielder and negative segregants still kept some dormancy with 75.8% ± 5.1 SE, 61.7% ± 5.3 SE, and 83.3% ± 4.4 SE at 10 days of imbibition, respectively (Figure 5A). The Bx17A1OE lines germinated faster and exhibited a lower dormancy with coleorhiza emergence (CE) of approximately 100% at 7 days of imbibition (Figure 5A). The addition of 1.5 mM acarbose, an allosteric inhibitor of α-amylase, did not restore grain dormancy in the Bx17A1OE lines (Supplementary Figure 8A). After 4 weeks of after-ripening, grains from both Fielder and the negative segregants released some dormancy and germinated faster, reaching 100% CE at 8 DPI while the Bx17A1OE lines reached 100% CE at 6 DPI (Figure 5B). The half grains with embryo released all dormancy and rapidly germinated to 100% CE in all negative controls (Fielder and negative segregants) and the Bx17A1OE lines (Figure 5C). When most of the α-amylase activity was inhibited by the addition of 1.5 mM acarbose, grain dormancy was not restored in Fielder or the negative segregants (Figures 5D,F). The addition of ABA could restore grain dormancy. All of the Bx17A1OE lines and negative controls were insensitive to the addition of 50 μM ABA in the medium (Supplementary Figures 8B,C). Germination of Fielder and 2-8NS was inhibited by 75 μM of ABA with CE of 61.6 and 77.8%, respectively (Figures 5C,E). Though the germination of another negative segregant (4-6NS) was slower, it had a relatively high CE with 88.8% ± 1.92 SE (Figure 5F). The Bx17A1OE lines were still insensitive to 150 μM of ABA and displayed high CE with 86.7–96.8% (Supplementary Figures 8B,C).

### Starch Degradation During Grain Germination

**FIGURE 6:**
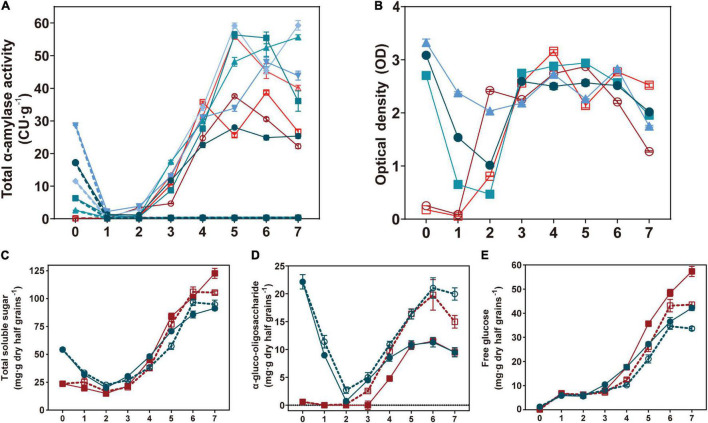
Effects on α-amylase activity TaAMY1 accumulation and total soluble sugar composition during germination. Total α-amylase activity **(A)**, TaAMY1 ELISA test **(B)**, total soluble sugar **(C)**, α-gluco-oligosaccharide **(D)**, and free glucose **(E)** were measured in half grains during germination. For **(A,B)**, different blue and red colors indicated five Bx17A1OE lines and negative controls, respectively. Plain and dash lines indicated absence or presence of acarbose. In **(C–E)**, the average of five Bx17A1OE lines (blue) and three negative (red) half grains during germination in the absence (plain) and presence of acarbose (dash) (1.5 mM) for 7 days. Values are expressed as mean ± SE.

To investigate the function of TaAMY1 during grain germination, total α-amylase activity, total soluble sugars, α-gluco-oligosaccharides, and free glucoses were measured during germination (Figure 6). In the absence of 1.5 mM acarbose and with the exception of the initial time point (high activities in Bx17A1OE lines), both Bx17A1OE lines and their controls showed no difference in their total α-amylase activities to 4 DPI. The highest α-amylase activities were detected after 5 DPI in the positive lines (45.3 CU.g^–1^ wholemeal on average) and negative controls (39.8 CU.g^–1^ wholemeal on average). In the presence of acarbose, the Bx17A1OE lines and controls showed an inhibition of total α-amylase activity and remained below 1 CU/g from 1 to 7 DPI (Figure 6A). The TaAMY1-specific LMA ELISA test on the same time points confirmed the presence of elevated but decreasing levels of TaAMY1 protein until 2 DPI (Figure 6B). After 3 DPI, there was no difference in the amount of TaAMY1 detected by the specific antibody between the Bx17AOE lines and the negative controls.

When total soluble sugars were investigated, positive lines showed a similar profile as negative controls with slightly higher sugar contents until 3–4 DPI (Figure 6B). The levels of total soluble sugars in the Bx17A1OE lines decreased from 54.2 mg.g^–1^ wholemeal (at imbibition) to 22.7 mg.g^–1^ wholemeal (2 DPI), then gradually increased up to 91.2 mg.g^–1^ wholemeal at 7 DPI (Figure 6C). The presence of acarbose did not appear to impact the accumulation of total soluble sugars except for a slight decrease from 4 DPI.

The elevated α-gluco-oligosaccharide levels present in the Bx17A1OE lines rapidly decreased during the first 2 DPI before reaching similar levels and profiles to those displayed by the negative controls, thus mirroring the trend seen for the total soluble sugars (Figure 6D). The presence of acarbose did not impact the reduction seen in the Bx17A1OE lines during the first 2 DPI. However, the addition of acarbose increased the overall α-gluco-oligosaccharide accumulation in both Bx17A1OE lines and controls (Figure 6D).

Finally, glucose accumulation was linear and similar in both Bx17A1OE and negative lines. The Bx17A1OE seems to accumulate smaller amount of glucose from 5 DPA. The presence of acarbose reduced the accumulation of glucose proportionally in both the Bx17A1OE lines and negative controls (Figure 6E).

## Discussion

The generation of wheat lines overexpressing TaAMY1 in grain had several objectives, including: (1) to determine the impact of elevated levels of TaAMY1 activity on starch structure and flour properties and (2) to examine the impact of elevated levels of TaAMY1 during grain development and subsequent grain germination compared to wild-type. It also provided the opportunity to compare the results obtained with overexpressed TaAMY1 to the previously published overexpressed TaAMY2 in the same background (Zhang et al., 2021).

To assess the impact of TaAMY1 accumulation on starch structure and properties in the developing grains, the high molecular glutenin promoter was selected for its grain specificity according to Oszvald et al. (2007b). However, increases of TaAMY1 proteins and relative activity in leaves and stems were detected, indicating some leakage of the promoter activity to other tissues, as was also observed by Whan et al. (2014). Nevertheless, similarly to what was described in Zhang et al. (2021), the elevated α-amylase activity did not have any impact on transitory starch or soluble sugar levels in leaves, suggesting the inability for the ectopically expressed enzymes to reach the chloroplast-located carbohydrates.

The several independent transgenic events offered a broad range of LMA-like responses, including ELISA detection signals and TaAMY1 α-amylase activity levels equal or superior to the activity seen in LMA-affected lines. In the Bx17A1OE grains, the highest α-amylase total activity was measured in the starchy endosperm. Activity was also detected to various degrees in the aleurone layer and embryo, but generally at a lower level than in the endosperm. There was no significant increase of total α-amylase in the husk of the Bx17A1OE grains. This was consistent with Bx17 being a high molecular weight (HMW) endosperm-specific promoter at least in the grain (Oszvald et al., 2007a; Wiley et al., 2007). In the adjacent aleurone layer, the relatively high TaAMY1 accumulation and α-amylase-detected activity might at least arise from the residual starchy endosperm tissue left during the grain dissection. However, this result is consistent with the findings of Mrva and Mares (1996) and Mares and Mrva (2014) describing high pI α-amylase synthesis around 20–30 DPA, in the aleurone layer, and, to a lesser extent, in the adjacent starchy endosperm of LMA wheat. The elevated levels of α-amylase activity in the Bx17A1OE grains impacted starch granules at the later stage of the grain development after 24 DPA despite the expression and production of an engineered and enzymatically active TaAMY1 from 10 to 15 DPA. It is plausible, as described in Zhang et al. (2021), that the TaAMY1 was not in direct contact with the starch until very late in the grain development when the grain starts desiccating and the amyloplast membranes become porous (Pepler et al., 2006). The absence of a TaAMY1 plastid targeting signal in the sequence, when analysed by the peptide prediction software ChloroP (Emanuelsson et al., 1999), tends to confirm this hypothesis. The TaAMY1 activity was sufficient to significantly decrease the number of B-type granules and increase the level of starch damage. Reserve starch is usually composed of large A-type granules (over 10 μM diameter and representing over 75% of the total granule area but only 15% of the total granule population on average) and small B-type granules (below 7 μM on average, representing around 80% of the population and 20% of the surface area) (Stoddard, 1999). During wheat grain development, A-type granules are generated during the first week post-anthesis while B-type-granules are generated only when the cellularisation has stopped, after the second week post anthesis (Parker, 1985). Studies showed that B-type granules have a different inner structure than A-type, including amylopectin chain length distribution, crystallinity, and microstructure that could facilitate digestion by TaAMY1 (Kim, 2009; Salman et al., 2009). This could indicate that B-type is more susceptible to degradation as a result of synthesis timing and/or structure.

The increased level of starch damage in dry grain reached up to 8% in some Bx17A1OE lines. However, this elevated level of starch damage did not directly impact the starch visco-properties. DSC was not impacted and the RVA profiles of the Bx17A1OE lines were almost fully restored by the addition of amylase inhibitor. This confirmed the very limited impact of any potential excess of TaAMY1 in the starch endosperm during grain development on gelatinization and visco-properties. It was also interesting to notice that a similar impact of amylase inhibitor on the RVA properties was described when TaAMY2 and TaAMY3 were overexpressed, but the recovery rate was lower, particularly for TaAMY2 (around 60%) (Whan et al., 2014; Zhang et al., 2021). This suggests different effects of the overexpression of TaAMY1 and TaAMY2 during grain development and germination.

Overexpressing TaAMY1 in the same genetic background used by Zhang et al. (2021) and describing overexpression of TaAMY2 allowed the comparison of the respective impacts of each α-amylase on grain composition, grain dormancy, and starch degradation during germination. While different promoters were used, the expression and activity profiles were very similar. This allowed proper comparison on each enzyme *modus operandi* on reserve starch degradation. The Bx17A1OE lines showed total soluble sugars at elevated levels, including free glucose, sucrose, and α-gluco-oligosaccharides. This was very similar to the degradation products of TaAMY2 and TaAMY3 overexpression, though the soluble sugar levels were lower than for TaAMY2 overexpression (Whan et al., 2014; Zhang et al., 2021). Thus, confirming the lesser direct impact of TaAMY1 on starch granule degradation. While wheat α-amylases have a lot of structural similarity inherent to the Glycosyl Hydrolases GH13 family (Janecek et al., 2014), TaAMY1 and TaAMY2 show different affinity or efficiency depending on the presence, the amount, the type of carbohydrate binding domain (CBM) or specific domain called the “Sugar Tong” (Janecek et al., 2014). For example, the TaAMY2 shows the presence of an extra sugar binding domain called SBS2 (Robert et al., 2003). This second starch branching enzyme (SBS) is described to be instrumental in lowering the enzyme *K*_*m*_, thus increasing the affinity for the glucan substrate and enhancing enzymatic activity (Nielsen et al., 2009). The lesser impact of TaAMY1 on starch granules could be explained by the absence of the extra SBS.

The Bx17A1OE lines showed a very low dormancy level compared to the negative controls, supporting the results previously found for TaAMY2. This low dormancy was associated with a strong resistance to ABA when half grains were imbibed on high level of ABA concentration. This lack of dormancy and ABA resistance can be attributed to the elevated levels of soluble sugars (De Laethauwer et al., 2013). When the starch degradation pathway is altered by engineering starch-related enzymes, such as overexpression of starch debranching enzyme or other isoforms of α-amylase, the derived accumulation of soluble sugar can overcome ABA inhibition (Du et al., 2018; Zhang et al., 2021). The lower level of soluble sugar present in Bx17A1OE lines compared to TaAMY2 overexpressed lines might explain the weaker ABA resistance seen in the Bx17A1OE lines. The use of acarbose and the inhibition of total α-amylase activity had no visible effect on the germination rate, which questions the importance of TaAMY1 and, generally, the importance of starch degradation by α-amylases on the early stage of germination. TaAmy1 expression peaks at 2–4 DPI (Mieog et al., 2017), and the absence of total α-amylase activity had no impact on germination and sugar accumulation for the first 10 days following imbibition. According to Nonogaki et al. (2010), and following the uptake of water (imbibition), the seed goes through a range of metabolic changes contributing to seedling emergence. Stored during the grain development, lipids and oligosaccharides in the bran and embryo are thought to serve as the initial fuel for germination (Clarke et al., 1983), while sugars are later mobilised to feed the respiration pathway (Plaxton, 1996). Following germination, seedling establishment is driven by the mobilisation of other seed storage compounds including starch and proteins. It is plausible that the degradation of starch by α-amylase is not immediately required but is more a prerequisite for future seedling establishment. Considering that germination *sensu stricto* begins with the uptake of water, imbibition, and includes the processes which occur prior to, and culminating in, coleorhiza emergence (Nonogaki et al., 2010), it is reasonable to assume that the absence of α-amylase has no impediment on germination. However, the impact of total α-amylase activity inhibition on coleoptile and coleorhizae growth would need to be assessed. The use of acarbose had a similar impact on sugar accumulation during germination to those described in Zhang et al. (2021) and included a slight reduction of total soluble sugar and free glucose along with a strong increase of gluco-oligosaccharides. Alpha-amylase action on starch does not directly lead to free glucose release. The role of α-amylase is to break down starch granules into smaller branched polysaccharides that become substrates for other degrading enzymes including β-amylase, iso-amylase, etc. (Lloyd et al., 2005). While we can apprehend the indirect effect of α-amylase inhibition on total soluble sugar reduction, the accumulation of gluco-oligosaccharide is intriguing as its accumulation when α-amylase is inhibited would imply gluco-oligosaccharide to be α-amylase substrate. We cannot rule out the possibility of acarbose having an inhibitory effect on alternative sources of carbohydrate degrading enzymes. The major involvement of α-amylase in gluco-oligosaccharide degradation needs to be considered. In addition, the consistent reduction of α-gluco-oligosaccharide in the first 2 days post-imbibition coupled with the normal accumulation of soluble sugar in the absence of active α-amylase suggests the presence of acarbose-resistant degrading enzymes active in the early stage of grain imbibition. Alternative amylolytic enzymes, such as β-amylase, isoamylase, or starch phosphorylase, could be at play to partially compensate for the lack of α-amylase. Sun and Henson (1990) and Henson and Sun (1995) followed by Sissons and MacGregor (1994) demonstrated that α-glucosidase was also capable of initiating starch degradation *in vitro* and that during the early stages of raw starch hydrolysis in germinating seeds, α-glucosidase is the second most important enzyme after α-amylase. Very recently, transcriptomic study of barley grain germination showed the presence of multiple degrading enzymes from 24 h post-imbibition. Developing an omic approach on germinating grain in presence or absence of acarbose would allow a complete understanding on the mechanistic in play.

The impact of the overexpressed TaAMY1 on germination was very limited. Both the activity and proteins generated by the transgene dropped quickly from the first day of imbibition within the first 24–48 h. The activity, rising after 48 h, seemed to be related to the endogenous and neosynthesized TaAMY1. Therefore, except for the initial elevated level of total soluble sugar (mainly gluco-oligosaccharides), the impact of the increased TaAMY1 on starch degradation during the early stage of germination is relatively limited. It was noticeable that not only the activity but also the ELISA signal decreased, suggesting a rapid loss of TaAMY1 protein integrity. Potential presence of proteases activated at the early stage of imbibition has been suggested previously. In cereal grains, several proteases have been shown to be involved in the germination process (Müntz et al., 2002; Grudkowska and Zagdańska, 2004; Tan-Wilson and Wilson, 2012; Szewinska et al., 2016). In wheat and barley, the cysteine proteases are the main proteases involved (de Barros and Larkins, 1994; Zhang and Jones, 1995; Bottari et al., 1996). There is no doubt that the activity generated by the excess of TaAMY1 has a major effect *in vitro* during the quality assessment. However, if proteases are present and responsible for rapid degradation of any enzymatic activity during grain imbibition, the impact of LMA-accumulating TaAMY1 on starch quality in the grain becomes questionable. The real impact of excess of grain specific TaAMY1 on baking quality and performance could provide additional information to this important question.

## Data Availability Statement

The data presented in the study are deposited in the data, CSIRO research data repository and it is now available following that link https://doi.org/10.25919/q0ej-v625.

## Author Contributions

QZ and JP were involved in the design and coordination of the study. They also conceived and performed experiments, analysed data, and wrote the manuscript. J-PR and J-RW were involved in the design and coordination of the study, conceived the experiments, analysed data, and wrote the manuscript. JM generated biological material and generated and analysed data related to the early generation and validation of TaAMY1 lines. MC and KB performed experiments and analysed data related to the mass spectrometry assessment. All authors read and approved the manuscript.

## Conflict of Interest

The authors declare that the research was conducted in the absence of any commercial or financial relationships that could be construed as a potential conflict of interest.

## Publisher’s Note

All claims expressed in this article are solely those of the authors and do not necessarily represent those of their affiliated organizations, or those of the publisher, the editors and the reviewers. Any product that may be evaluated in this article, or claim that may be made by its manufacturer, is not guaranteed or endorsed by the publisher.
