# Supplementary material for: Over-Expression of a Wheat Late Maturity Alpha-Amylase Type 1 Impact on Starch Properties During Grain Development and Germination
Source: Front Plant Sci. 2022 Mar 29;13:811728. doi: 10.3389/fpls.2022.811728 (PMC9002352; doi:10.3389/fpls.2022.811728)
Supplement: Supplementary file 1 [file Data_Sheet_1.docx]

# Supplementary Figures


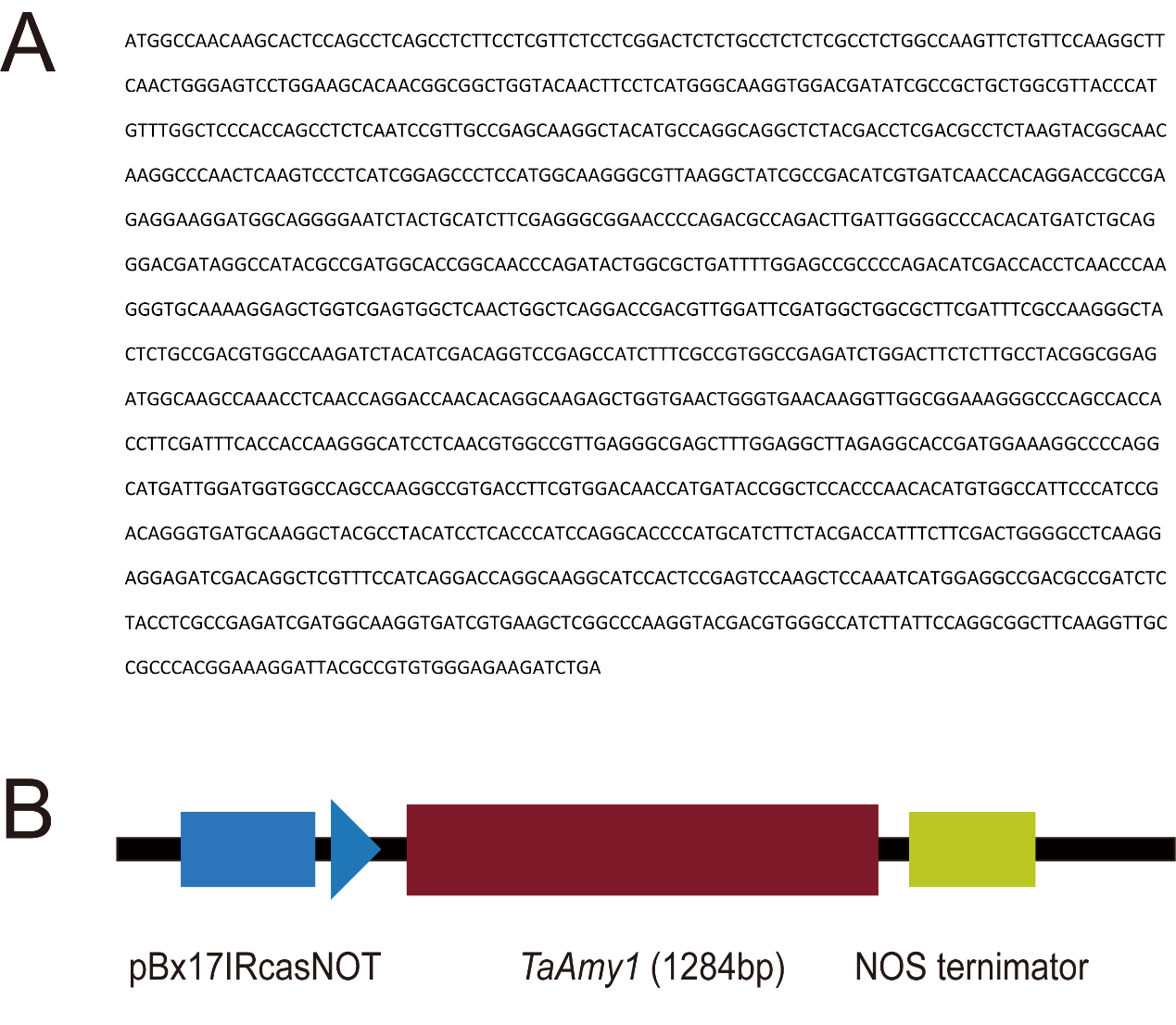


**Figure S1.** Over-expression TaAMY1 construct driven by Bx17 promoter. (A) is the CDS of *TaAmy1* gene and (B) is the schematic diagram of construct.


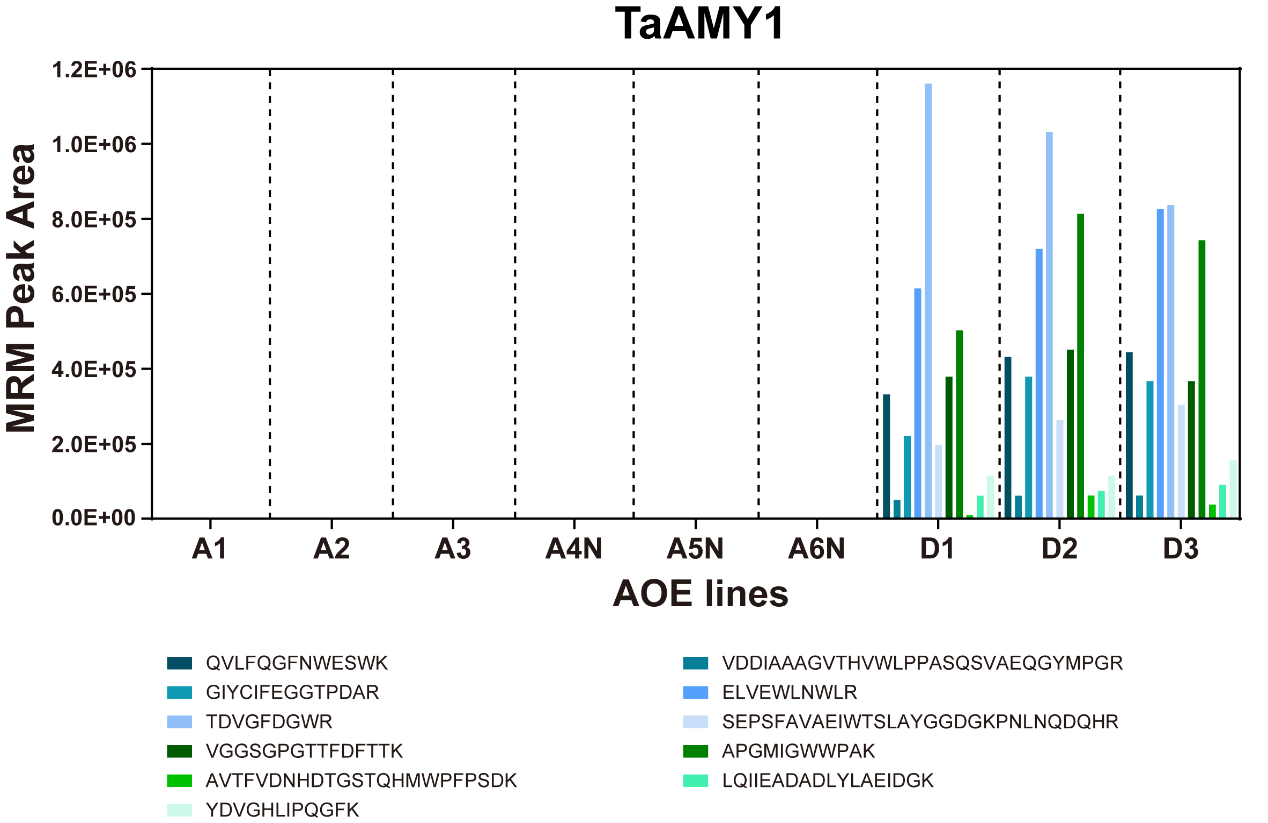


**Figure S2.** Relative quantitation and comparison of TaAMY1 peptides using mass spectrometry. Proteins were extracted from mature grain of Bx17A1OE-4 (D1, D2, D3) and digested using trypsin. The UA2OE lines (A1, A2 and A3) and their isogenic negative controls (A4N, A5N and A6N) were included as controls, proving that the method detects TaAMY1 and not TaAMY2. The LC-MRM-MS peak area for each of the 11 identified peptides are plotted in the order in which they appear in the TaAMY1 sequence. The MRM peak area is summed peak area of three MRM transitions.


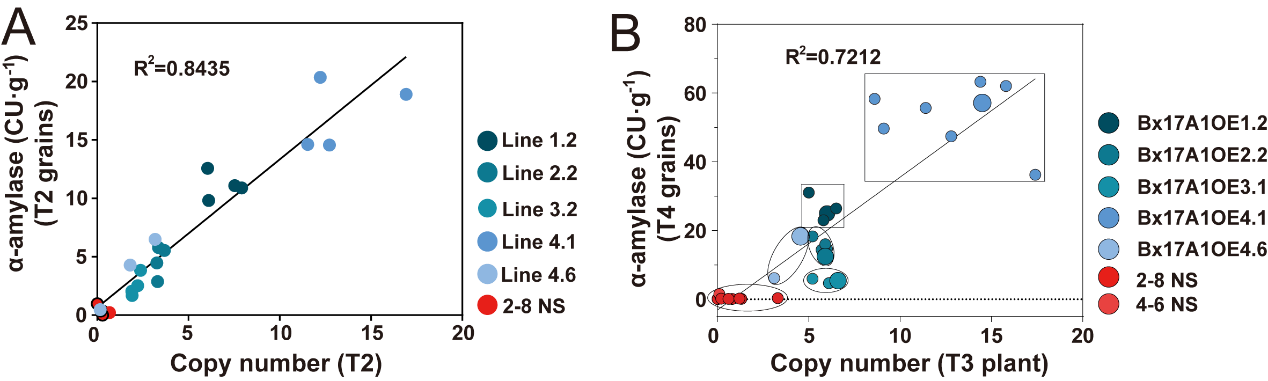


**Figure S3.** The correlation between copy number and total α-amylase activity. **A** displayed correlation between copy number (T2) and α-amylase activity (T2 grains). **B** displayed correlation between copy number (T3 plants) and α-amylase activity (T4 mature grains). The same dots were from one line, and the bigger one was selected for deeper analysis.


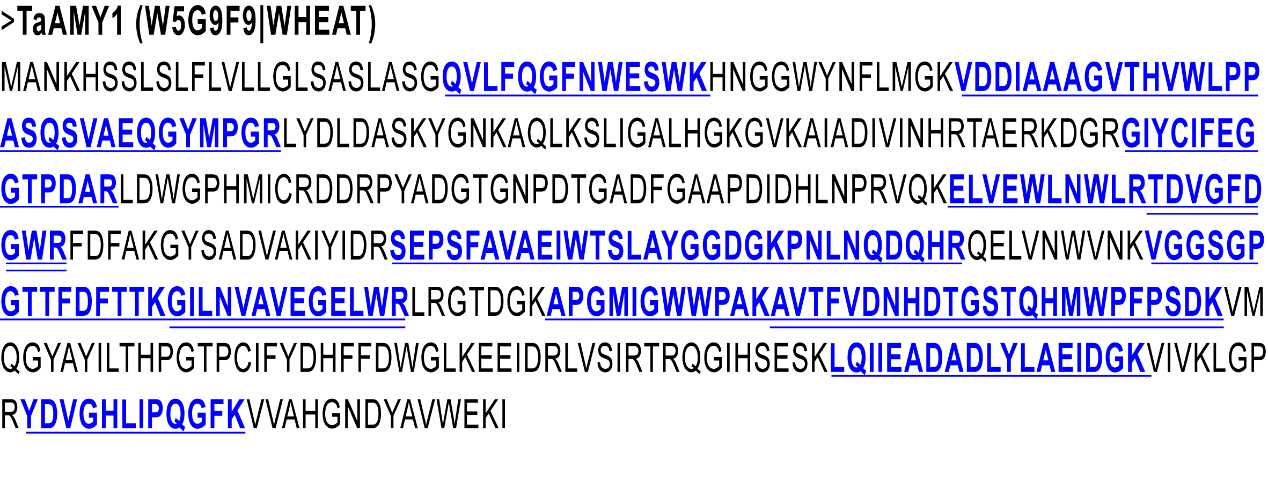


**Figure S4**. TaAMY1 sequence with mapped tryptic peptides (blue bold, underlined). Peptides selected for sMRM monitoring were fully tryptic, i.e. contained no missed cleavages (double underlined). As some of the peptides were adjacent in the sequence, these have been differentiated by single or double underline.


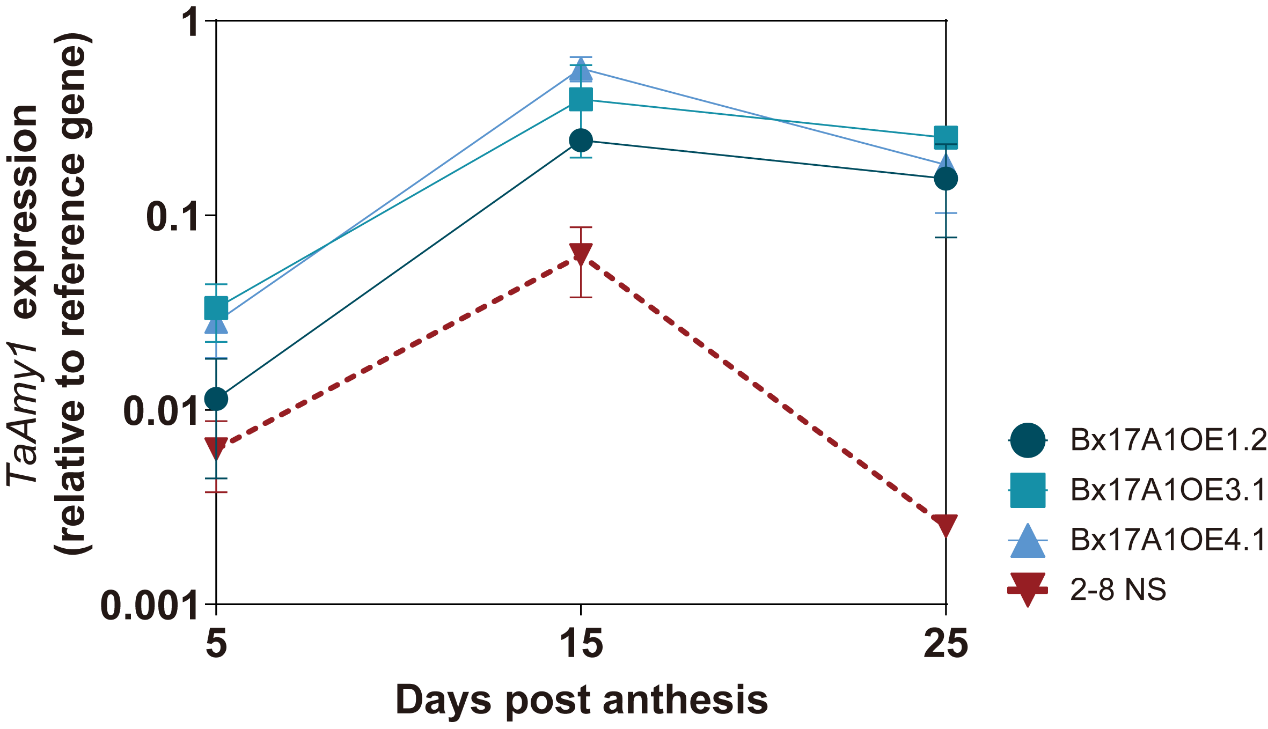


**Figure S5.** TaAmy1 expression in developing grains. *TaAmy1* transcript level was relative to geometric mean of *TaActin*, *Ta.14126.1.S1_at* and *Ta.7894.3.A1_at* at seed development in whole grain

.


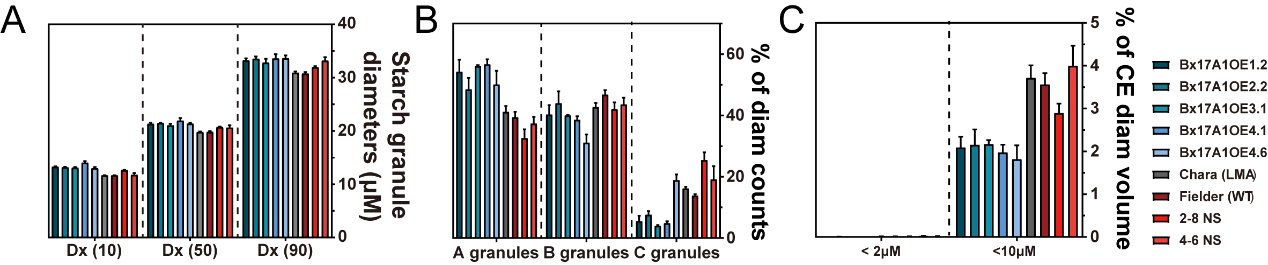


**Figure S6.** Effect on starch granule distribution. (A) is starch granules distribution of the whole starch granules, (**B**) represents the circular equivalent CE diam counts of A, B and C granules and (**C**) is CE diam volume of starch granules below 10μM. Dx (10, 50 and 90) represents the diameter of starch granules when the cumulative distribution of particles diameter is 10%; 50% and 90% respectively. The granules of diam below 2μm were C granules; The granules of diam between 2 to 10μm were B granules; The granules of diam over 10μm were A granules. Blue, grey and red indicated five Bx17A1OE lines, Chara (LMA) and three negative controls, respectively.


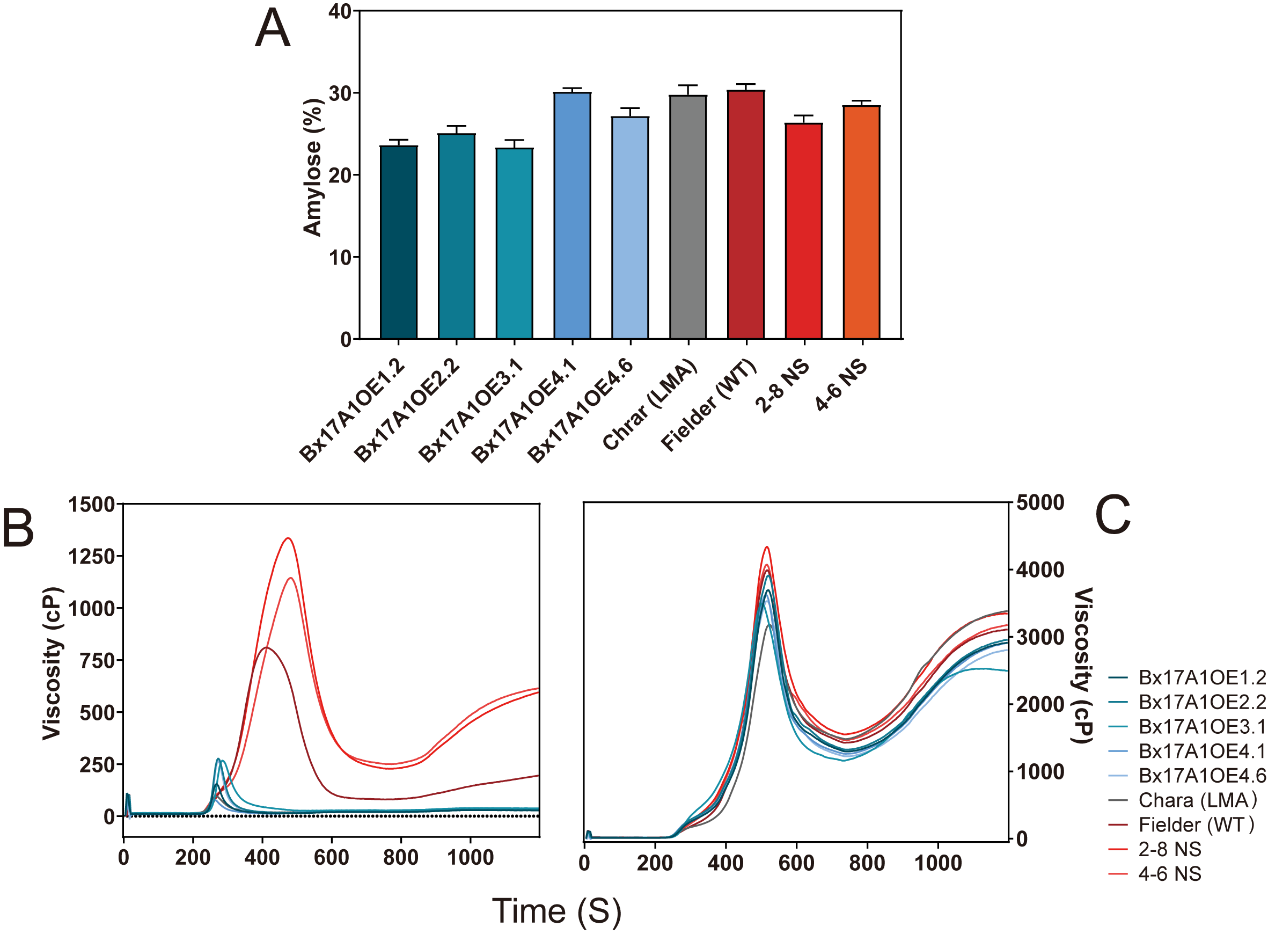


**Figure S7**. Effect on amylose content and RVA. (A) represents the amylose content in purified starch. Rapid Visco Analyser comparison among five positive lines, three negative lines and Chara wholemeal in absence of silver nitrate (**B**) and presence of silver nitrate (**C**). Blue, grey and red indicated five Bx17A1OE lines, Chara (LMA) and three negative controls, respectively

.


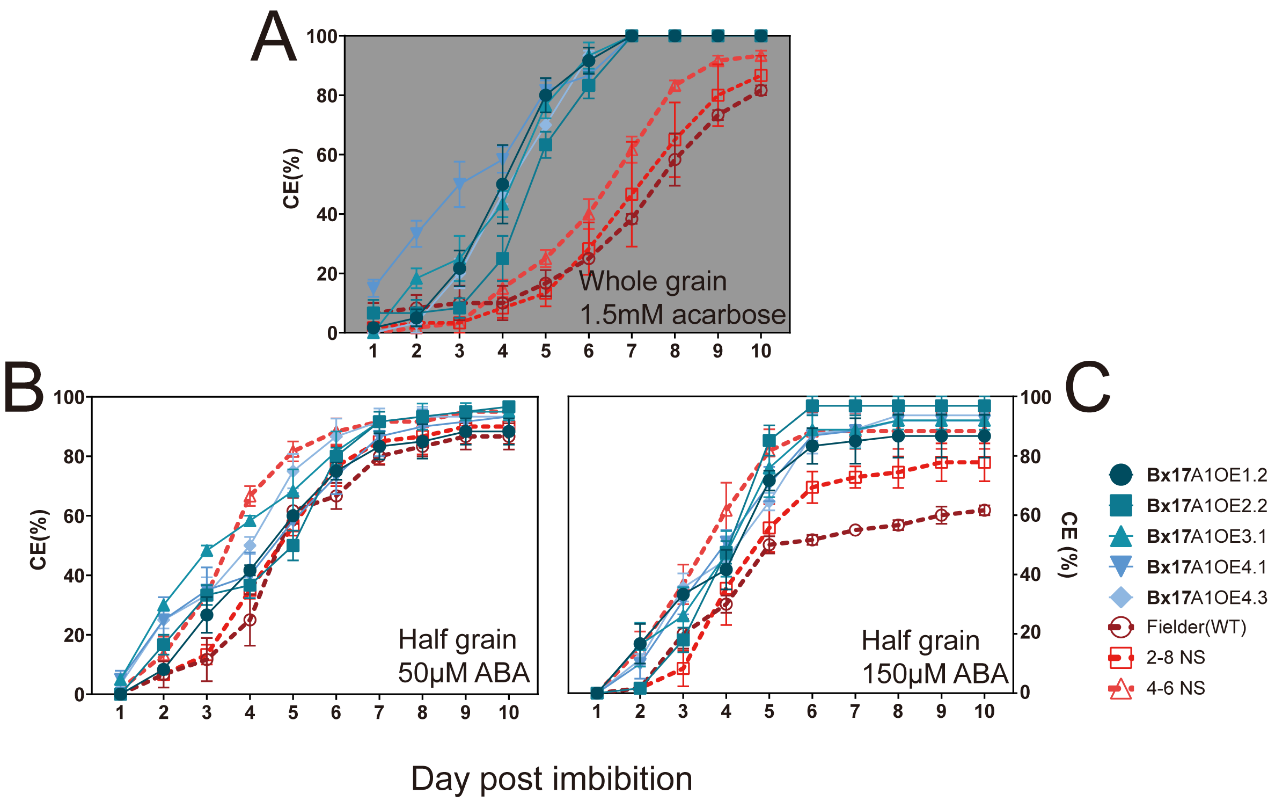


**Figure S8.** Effect of abscisic acid on germination. Coleorhiza emergence (CE) of whole grains with 1.5mM acarbose (**A**) or half grains with additional of 50μM ABA (**B**) and 150μM ABA (**C**)

**Supplementary Tables**

**Table S1.** Primer pairs used for copy number (qPCR) or gene expression (RT-qPCR).

| **PCR type** | **Target gene** | **Description** | **Forward** | **Reverse** | **Length (bp)** | **productTm (℃)** |
| --- | --- | --- | --- | --- | --- | --- |
| qPCR | EC A | Reference gene | ACCTGACCTTGTAAAACCATTCAT | TGACATCCTCCAACATCTCTAAC | 104 | 79 |
| qPCR | NOS | Target gene for copy number | TTGAATCCTGTTGCCGGTCT | GCGGGACTCTAATCATAAAAACCC | 127 | 78 |
| RT-qPCR | TaActin | Reference gene | TCAGCCGAGCGGGAAATTGT | CCTCTCTGCGCCAATCGT | 156 | 88 |
| RT-qPCR | Ta.14126.1.S1_at | Reference gene | GAGTCTGCCCACCCATTCGTAA | GACATGCCATAGGTTTCAGCGAC | 155 | 82 |
| RT-qPCR | Ta.7894.3.A1_at | Reference gene | AGCAAGTTGTGACCCGAGGA | GGCGTCAGCAAATAGCAAGTG | 73 | 83 |
| RT-qPCR | TaAMY1 | Gene expression | AGCTCGTCGAGTGGCTCAACTG | AGCGACGTCCATATCTCGGC | 151 | 89 |

**Table S2**. Wheat α-amylase lines and method of protein extraction.

| Code | Name | Weight | Description | Variety |
| --- | --- | --- | --- | --- |
| A1 | UA2OE | 3 seeds | Alpha-amylase type 2 over-expression using ubiquitin promoter. Wholemeal flour (mature grains crushed); 3 replicates. | Fielder |
| A2 | UA2OE | 3 seeds |  |  |
| A3 | UA2OE | 3 seeds |  |  |
| A4N | UA2NS | 3 seeds | UA2OE negative segregants negative control. Wholemeal flour (mature grains crushed); 3 replicates. |  |
| A5N | UA2NS | 3 seeds |  |  |
| A6N | UA2NS | 3 seeds |  |  |
| D1 | Bx17A1OE-4 | 3 seeds | Alpha-amylase type 1 overexpression using Ubiquitin promoter. Wholemeal flour (mature grains crushed); 3 replicates. |  |
| D2 | Bx17A1OE-4 | 3 seeds |  |  |
| D3 | Bx17A1OE-4 | 3 seeds |  |  |

**Table S3.** Multiple reaction monitoring transitions of TaAMY1 peptides.

| **Protein** | **Peptide^a^** | **RT (min)^b^** | **Q1** | **z ^b^** | **Q3** | **Fragment** | **CE ^c^** |
| --- | --- | --- | --- | --- | --- | --- | --- |
|  |  |  | ***m/z* ^b^** |  | ***m/z* ^b^** |  |  |
| Ta AMY1 | QVLFQGFNWESWK | 8.8 | 834.91 | 2+ | 849.39 | y6+ | 39.9 |
|  |  |  |  |  | 1053.48 | y8+ |  |
|  |  |  |  |  | 1181.54 | y9+ |  |
| Ta AMY1 | QVLFQGFNWESWK | 9.1 | 826.40 | 2+ | 849.39 | y6+ | 39.5 |
|  |  |  |  |  | 1053.48 | y8+ |  |
|  |  |  |  |  | 1181.54 | y9+ |  |
| Ta AMY1 | VDDIAAAGVTHVWLPPASQSVAEQGYMPGR | 6.8 | 781.39 | 4+ | 1448.75 | b14+ | 37.1 |
|  |  |  |  |  | 680.32 | y6+ |  |
|  |  |  |  |  | 1008.46 | y9+ |  |
| Ta AMY1 | GIYCIFEGGTPDAR | 5.8 | 778.36 | 2+ | 1062.521 | y10+ | 37.1 |
|  |  |  |  |  | 802.369 | y8+ |  |
|  |  |  |  |  | 949.437 | y9+ |  |
| Ta AMY1 | ELVEWLNWLR | 8.7 | 679.37 | 2+ | 701.409 | y5+ | 32.3 |
|  |  |  |  |  | 887.488 | y6+ |  |
|  |  |  |  |  | 1016.531 | y7+ |  |
| Ta AMY1 | TDVGFDGWR | 4.8 | 526.74 | 2+ | 533.247 | y4+ | 24.8 |
|  |  |  |  |  | 680.315 | y5+ |  |
|  |  |  |  |  | 737.336 | y6+ |  |
| Ta AMY1 | SEPSFAVAEIWTSLAYGGDGKPNLNQDQHR | 7.8 | 822.65 | 4+ | 849.901 | y15++ | 39.1 |
|  |  |  |  |  | 885.419 | y16++ |  |
|  |  |  |  |  | 1121.545 | y9+ |  |
| Ta AMY1 | VGGSGPGTTFDFTTK | 4.8 | 736.36 | 2+ | 1114.542 | y10+ | 35.1 |
|  |  |  |  |  | 1171.563 | y11+ |  |
|  |  |  |  |  | 859.42 | y7+ |  |
| Ta AMY1 | GILNVAVEGELWR | 7.4 | 728.40 | 2+ | 789.389 | y6+ | 34.7 |
|  |  |  |  |  | 888.457 | y7+ |  |
|  |  |  |  |  | 959.495 | y8+ |  |
| Ta AMY1 | APGMIGWWPAK | 6.7 | 607.31 | 2+ | 744.383 | y6+ | 28.8 |
|  |  |  |  |  | 857.467 | y7+ |  |
|  |  |  |  |  | 1045.529 | y9+ |  |
| Ta AMY1 | APG**M**IGWWPAK | 6.0 | 615.31 | 2+ | 744.383 | y6+ | 29.2 |
|  |  |  |  |  | 857.467 | y7+ |  |
|  |  |  |  |  | 1061.524 | y9+ |  |
| Ta AMY1 | AVTFVDNHDTGSTQHMWPFPSDK | 5.8 | 655.05 | 4+ | 690.346 | y6+ | 30.8 |
|  |  |  |  |  | 876.425 | y7+ |  |
|  |  |  |  |  | 1007.466 | y8+ |  |
| Ta AMY1 | AVTFVDNHDTGSTQH**M**WPFPSDK | ND | 659.05 | 4+ | 690.346 | y6+ | 31 |
|  |  |  |  |  | 876.425 | y7+ |  |
|  |  |  |  |  | 1023.466 | y8+ |  |
| Ta AMY1 | LQIIEADADLYLAEIDGK | 7.7 | 664.02 | 3+ | 632.325 | y6+ | 29.9 |
|  |  |  |  |  | 745.409 | y7+ |  |
|  |  |  |  |  | 908.472 | y8+ |  |
| Ta AMY1 | YDVGHLIPQGFK | 5.7 | 687.36 | 2+ | 798.42 | b7+ | 32.70 |
|  |  |  |  |  | 576.31 | y5+ |  |
|  |  |  |  |  | 802.48 | y7+ |  |

1. The peptide sequence is represented by single amino acid code.
2. RT, retention time (min); Q1 *m/z*, precursor ion mass-to-charge ratio; z, charge state; Q3 *m/z*, fragment ion *m/z*; CE, collision energy in V. ND refers to not detected, which was the case for some of the methionine oxidation products (bold **M**).
3. Collision energy settings were calculated for 2+ ions (CE = slope (0.049) x (precursor *m/z*) + intercept (-1.0); 3+ ions (CE = slope (0.048) x (precursor *m/z*) + intercept (-2.0) and 4+ ions (CE = slope (0.050) x (precursor *m/z*) + intercept (-2.0).

**Table S4**. Copy number information in different generations. Copy number was performed by Real-time PCR. Two technical replicates were performed. In every generation, the plant in red font was planted in greenhouse for deeper analysis. In T2 and T3 generations, every plant harvested in separated bag. In T4 plants, copy number was checked in every plant

Table S4.1 The track of copy number of Bx17A1OE1.2 from T1 to T4.

| T1 | | T2 | | T3 | | T4 | |
| --- | --- | --- | --- | --- | --- | --- | --- |
| Plant | Copies | Plant | Copies | Plant | Copies | Plant | Copies |
| **1** | **8** | 1 | 6 |  |  |  |  |
|  |  | 2 | 6 |  |  |  |  |
|  |  | 3 | 7 |  |  |  |  |
|  |  | **4** | **6** | **1** | **6** | 1 | 6 |
|  |  |  |  |  |  | 2 | 7 |
|  |  |  |  |  |  | 3 | 7 |
|  |  |  |  |  |  | 4 | 7 |
|  |  |  |  |  |  | 5 | 9 |
|  |  |  |  |  |  | 6 | 8 |
|  |  |  |  |  |  | 7 | 7 |
|  |  |  |  |  |  | 8 | 7 |
|  |  |  |  | 2 | 7 |  |  |
|  |  |  |  | 3 | 7 |  |  |
|  |  |  |  | 4 | 6 |  |  |
|  |  |  |  | 5 | 6 |  |  |
|  |  |  |  | 6 | 8 |  |  |
|  |  |  |  | 7 | 7 |  |  |
|  |  |  |  | 8 | 7 |  |  |
|  |  |  |  | 9 | 8 |  |  |
|  |  |  |  | 10 | 7 |  |  |
|  |  |  |  | 11 | 7 |  |  |
|  |  |  |  | 12 | 6 |  |  |
|  |  |  |  | 13 | 6 |  |  |
|  |  |  |  | 14 | 6 |  |  |
|  |  |  |  | 15 | 6 |  |  |
|  |  |  |  | 16 | 6 |  |  |
|  |  |  |  | 17 | 6 |  |  |
|  |  |  |  | 18 | 7 |  |  |
|  |  |  |  | 19 | 6 |  |  |
|  |  |  |  | 20 | 6 |  |  |
|  |  |  |  | 21 | 5 |  |  |
|  |  |  |  | 22 | 5 |  |  |
|  |  |  |  | 23 | 5 |  |  |
|  |  |  |  | 24 | 7 |  |  |
|  |  | 5 | 6 |  |  |  |  |
|  |  | 6 | 7 |  |  |  |  |
|  |  | 7 | 7 |  |  |  |  |
|  |  | 8 | 9 |  |  |  |  |

**Table S4.2** The track of copy number of Bx17A1OE2.2 from T1 to T4.

| T1 | | T2 | | T3 | | T4 | |
| --- | --- | --- | --- | --- | --- | --- | --- |
| Plant | Copies | Plant | Copies | Plant | Copies | Plant | Copies |
| **2** | **4** | 1 | 5 |  |  |  |  |
|  |  | **2** | **4** | 1 | 5 |  |  |
|  |  |  |  | 2 | 5 |  |  |
|  |  |  |  | 3 | 3 |  |  |
|  |  |  |  | 4 | 5 |  |  |
|  |  |  |  | 5 | 4 |  |  |
|  |  |  |  | 6 | 3 |  |  |
|  |  |  |  | 7 | 3 |  |  |
|  |  |  |  | 8 | 3 |  |  |
|  |  |  |  | 9 | 3 |  |  |
|  |  |  |  | 10 | 3 |  |  |
|  |  |  |  | 11 | 6 |  |  |
|  |  |  |  | 12 | 5 |  |  |
|  |  |  |  | 13 | 5 |  |  |
|  |  |  |  | 14 | 6 |  |  |
|  |  |  |  | 15 | 4 |  |  |
|  |  |  |  | 16 | 6 |  |  |
|  |  |  |  | 17 | 4 |  |  |
|  |  |  |  | 18 | 3 |  |  |
|  |  |  |  | **19** | **4** | 1 | 4 |
|  |  |  |  |  |  | 2 | 4 |
|  |  |  |  |  |  | 3 | 5 |
|  |  |  |  |  |  | 4 | 4 |
|  |  |  |  |  |  | 5 | 5 |
|  |  |  |  |  |  | 6 | 4 |
|  |  |  |  |  |  | 7 | 4 |
|  |  |  |  |  |  | 8 | 4 |
|  |  |  |  | 20 | 6 |  |  |
|  |  |  |  | 21 | 6 |  |  |
|  |  |  |  | 22 | 4 |  |  |
|  |  |  |  | 23 | 5 |  |  |
|  |  |  |  | 24 | 6 |  |  |
|  |  | 3 | 3 |  |  |  |  |
|  |  | 4 | 4 |  |  |  |  |

**Table S4.3** The track of copy number of Bx17A1OE3.1 from T1 to T4.

| T1 | | T2 | | T3 | | T4 | |
| --- | --- | --- | --- | --- | --- | --- | --- |
| Plant | Copies | Plant | Copies | Plant | Copies | Plant | Copies |
| 1 | NA | 1 | 2 |  |  |  |  |
|  |  | **2** | **2** | 1 | 4 |  |  |
|  |  |  |  | 2 | 6 |  |  |
|  |  |  |  | 3 | 5 |  |  |
|  |  |  |  | 4 | 5 |  |  |
|  |  |  |  | 5 | 4 |  |  |
|  |  |  |  | 6 | 6 |  |  |
|  |  |  |  | 7 | 4 |  |  |
|  |  |  |  | 8 | 4 |  |  |
|  |  |  |  | 9 | 3 |  |  |
|  |  |  |  | 10 | 4 |  |  |
|  |  |  |  | 11 | 3 |  |  |
|  |  |  |  | 12 | 1 |  |  |
|  |  |  |  | 13 | 4 |  |  |
|  |  |  |  | 14 | 3 |  |  |
|  |  |  |  | 15 | 5 |  |  |
|  |  |  |  | 16 | 3 |  |  |
|  |  |  |  | 17 | 4 |  |  |
|  |  |  |  | **18** | **3** | 1 | 4 |
|  |  |  |  |  |  | 2 | 5 |
|  |  |  |  |  |  | 3 | 5 |
|  |  |  |  |  |  | 4 | 5 |
|  |  |  |  |  |  | 5 | 4 |
|  |  |  |  |  |  | 6 | 4 |
|  |  |  |  |  |  | 7 | 4 |
|  |  |  |  |  |  | 8 | 4 |
|  |  |  |  | 19 | 4 |  |  |
|  |  |  |  | 20 | 5 |  |  |
|  |  |  |  | 21 | 7 |  |  |
|  |  |  |  | 22 | 4 |  |  |
|  |  |  |  | 23 | 4 |  |  |
|  |  |  |  | 24 | 3 |  |  |
|  |  | 3 | 2 |  |  |  |  |
|  |  | 4 | 2 |  |  |  |  |

**Table S4.**4 The track of copy number of Bx17A1OE4.1 from T1 to T4.

| T1 | | T2 | | T3 | | T4 | |
| --- | --- | --- | --- | --- | --- | --- | --- |
| Plant | Copies | Plant | Copies | Plant | Copies | Plant | Copies |
| **1** | **16** | **1** | **16** | 1 | 14 |  |  |
|  |  |  |  | 2 | 14 |  |  |
|  |  |  |  | **3** | **15** | 1 | 12 |
|  |  |  |  |  |  | 2 | 14 |
|  |  |  |  |  |  | 3 | 13 |
|  |  |  |  |  |  | 4 | 14 |
|  |  |  |  |  |  | 5 | 14 |
|  |  |  |  |  |  | 6 | 11 |
|  |  |  |  |  |  | 7 | 11 |
|  |  |  |  |  |  | 8 | 11 |
|  |  |  |  |  |  | 9 | 14 |
|  |  |  |  |  |  | 10 | 14 |
|  |  |  |  |  |  | 11 | 11 |
|  |  |  |  |  |  | 12 | 11 |
|  |  |  |  |  |  | 13 | 14 |
|  |  |  |  |  |  | 14 | 11 |
|  |  |  |  |  |  | 15 | 10 |
|  |  |  |  |  |  | 16 | 12 |
|  |  |  |  |  |  | 17 | 14 |
|  |  |  |  |  |  | 18 | 17 |
|  |  |  |  |  |  | 19 | 17 |
|  |  |  |  |  |  | 20 | 16 |
|  |  |  |  |  |  | 21 | 13 |
|  |  |  |  |  |  | 22 | 16 |
|  |  |  |  |  |  | 23 | 16 |
|  |  |  |  |  |  | 24 | 14 |
|  |  |  |  |  |  | 25 | 15 |
|  |  |  |  |  |  | 26 | 14 |
|  |  |  |  |  |  | 27 | 18 |
|  |  |  |  |  |  | 28 | 16 |
|  |  |  |  | 4 | 14 |  |  |
|  |  |  |  | 5 | 14 |  |  |
|  |  |  |  | 6 | 13 |  |  |
|  |  |  |  | 7 | 12 |  |  |
|  |  |  |  | 8 | 11 |  |  |
|  |  |  |  | 9 | 9 |  |  |
|  |  |  |  | 10 | 11 |  |  |
|  |  |  |  | 11 | 13 |  |  |
|  |  |  |  | 12 | 13 |  |  |
|  |  |  |  | 13 | 11 |  |  |
|  |  |  |  | 14 | 12 |  |  |
|  |  |  |  | 15 | 12 |  |  |
|  |  |  |  | 16 | 13 |  |  |
|  |  |  |  | 17 | 13 |  |  |
|  |  |  |  | 18 | 15 |  |  |
|  |  |  |  | 19 | 11 |  |  |
|  |  |  |  | 20 | 12 |  |  |
|  |  |  |  | 21 | 14 |  |  |
|  |  |  |  | 22 | 15 |  |  |
|  |  |  |  | 23 | 16 |  |  |
|  |  |  |  | 24 | 17 |  |  |
|  |  | 2 | 17 |  |  |  |  |
|  |  | 3 | 18 |  |  |  |  |
|  |  | 4 | 12 |  |  |  |  |
|  |  | 5 | 12 |  |  |  |  |
|  |  | 6 | 14 |  |  |  |  |
|  |  | 7 | 13 |  |  |  |  |
|  |  | 8 | 11 |  |  |  |  |
|  |  | 9 | 13 |  |  |  |  |
|  |  | 10 | 8 |  |  |  |  |
|  |  | 11 | 15 |  |  |  |  |
|  |  | 12 | 11 |  |  |  |  |

**Table S4**.5 The track of copy number of Bx17A1OE4.6 from T1 to T4.

| T1 | | T2 | | T3 | | T4 | |
| --- | --- | --- | --- | --- | --- | --- | --- |
| Plant | Copies | Plant | Copies | Plant | Copies | Plant | Copies |
| **1** | **2** | 1 | 0 |  |  |  |  |
|  |  | 2 | 0 |  |  |  |  |
|  |  | 3 | 0 |  |  |  |  |
|  |  | **4** | **4** | 1 | 0 |  |  |
|  |  |  |  | 2 | 3 |  |  |
|  |  |  |  | 3 | 3 |  |  |
|  |  |  |  | 4 | 0 |  |  |
|  |  |  |  | 5 | 0 |  |  |
|  |  |  |  | **6** | **5** | 1 | 6 |
|  |  |  |  |  |  | 2 | 4 |
|  |  |  |  |  |  | 3 | 4 |
|  |  |  |  |  |  | 4 | 5 |
|  |  |  |  |  |  | 5 | 4 |
|  |  |  |  |  |  | 6 | 5 |
|  |  |  |  |  |  | 7 | 4 |
|  |  |  |  |  |  | 8 | 4 |
|  |  |  |  |  |  | 9 | 5 |
|  |  |  |  |  |  | 10 | 4 |
|  |  |  |  |  |  | 11 | 3 |
|  |  |  |  |  |  | 12 | 4 |
|  |  |  |  |  |  | 13 | 4 |
|  |  |  |  |  |  | 14 | 3 |
|  |  |  |  |  |  | 15 | 5 |
|  |  |  |  |  |  | 16 | 4 |
|  |  |  |  |  |  | 17 | 4 |
|  |  |  |  |  |  | 18 | 4 |
|  |  |  |  |  |  | 19 | 3 |
|  |  |  |  |  |  | 20 | 4 |
|  |  |  |  |  |  | 21 | 3 |
|  |  |  |  |  |  | 22 | 5 |
|  |  |  |  |  |  | 23 | 4 |
|  |  |  |  |  |  | 24 | 4 |
|  |  |  |  | 7 | 3 |  |  |
|  |  |  |  | 8 | 0 |  |  |
|  |  |  |  | 9 | 2 |  |  |
|  |  |  |  | 10 | 1 |  |  |
|  |  |  |  | 11 | 0 |  |  |
|  |  |  |  | 12 | 2 |  |  |
|  |  |  |  | 13 | 3 |  |  |
|  |  |  |  | 14 | 0 |  |  |
|  |  |  |  | 15 | 2 |  |  |
|  |  |  |  | 16 | 3 |  |  |
|  |  |  |  | 17 | 2 |  |  |
|  |  |  |  | 18 | 2 |  |  |
|  |  |  |  | 19 | 0 |  |  |
|  |  |  |  | 20 | 2 |  |  |
|  |  |  |  | 21 | 0 |  |  |
|  |  |  |  | 22 | 5 |  |  |
|  |  |  |  | 23 | 2 |  |  |
|  |  |  |  | 24 | 3 |  |  |
|  |  | 5 | 3 |  |  |  |  |
|  |  | 6 | 3 |  |  |  |  |
|  |  | 7 | 2 |  |  |  |  |
|  |  | 8 | 2 |  |  |  |  |
|  |  | 9 | 2 |  |  |  |  |
